# SlWRKY45 interacts with jasmonate-ZIM domain proteins to negatively regulate defense against the root-knot nematode *Meloidogyne incognita* in tomato

**DOI:** 10.1093/hr/uhac197

**Published:** 2022-09-05

**Authors:** Huang Huang, Wenchao Zhao, Hui Qiao, Chonghua Li, Lulu Sun, Rui Yang, Xuechun Ma, Jilin Ma, Susheng Song, Shaohui Wang

**Affiliations:** Plant Science and Technology College, Beijing University of Agriculture, Beijing, 102206, China; Beijing Key Laboratory for Agricultural Application and New Technique, Beijing University of Agriculture, Beijing, 102206, China; Plant Science and Technology College, Beijing University of Agriculture, Beijing, 102206, China; Beijing Key Laboratory for Agricultural Application and New Technique, Beijing University of Agriculture, Beijing, 102206, China; Plant Science and Technology College, Beijing University of Agriculture, Beijing, 102206, China; Plant Science and Technology College, Beijing University of Agriculture, Beijing, 102206, China; Plant Science and Technology College, Beijing University of Agriculture, Beijing, 102206, China; Beijing Key Laboratory for Agricultural Application and New Technique, Beijing University of Agriculture, Beijing, 102206, China; Beijing Key Laboratory for Agricultural Application and New Technique, Beijing University of Agriculture, Beijing, 102206, China; Plant Science and Technology College, Beijing University of Agriculture, Beijing, 102206, China; Plant Science and Technology College, Beijing University of Agriculture, Beijing, 102206, China; College of Life Sciences, Capital Normal University, Beijing, 100048, China; Plant Science and Technology College, Beijing University of Agriculture, Beijing, 102206, China; Beijing Key Laboratory for Agricultural Application and New Technique, Beijing University of Agriculture, Beijing, 102206, China

## Abstract

Parasitic root-knot nematodes (RKNs) cause a severe reduction in crop yield and seriously threaten agricultural production. The phytohormones jasmonates (JAs) are important signals regulating resistance to multiple biotic and abiotic stresses. However, the molecular mechanism for JAs-regulated defense against RKNs in tomato remains largely unclear. In this study, we found that the transcription factor SlWRKY45 interacted with most JA-ZIM domain family proteins (JAZs), key repressors of the JA signaling. After infection by the RKN *Meloidogyne incognita*, the *slwrky45* mutants exhibited lower gall numbers and egg numbers per gram of roots than wild type, whereas overexpression of *SlWRKY45* attenuated resistance to *Meloidogyne incognita*. Under *M. incognita* infection, the contents of jasmonic acid (JA) and JA-isoleucine (JA-Ile) in roots were repressed by *SlWRKY45-*overexpression. Furthermore, SlWRKY45 bound to and inhibited the promoter of the JA biosynthesis gene *ALLENE OXIDE CYCLASE* (*AOC*), and repressed its expression. Overall, our findings revealed that the SlJAZ-interaction protein SlWRKY45 attenuated RKN-regulated JA biosynthesis and repressed defense against the RKN *M. incognita* in tomato.

## Introduction

The phytoparasitic root-knot nematodes (RKNs *Meloidogyne* spp.) are extensively distributed throughout the world. Among them, *Meloidogyne incognita* is known as one of the most harmful RKNs [1]. Infective juveniles (J2s) of RKNs infect roots of plants, parasitize vascular cylinders, and stimulate roots to develop giant cells and form root-knots (galls) [2–4]. These galls destroy the normal physiological activities of the roots, hinder transport of water and nutrients, reduce host growth and yield, and even lead to host plant death [5, 6].

Jasmonates (JAs), a class of phytohormones, comprise jasmonic acid (JA) and its derivatives including jasmonic acid-isoleucine (JA-Ile), and methyl-jasmonic acid (MeJA) [7]. The study of JA signal transduction began with the screening of *Arabidopsis* coronatine (a JA-Ile mimic)-insensitive mutants and cloning of COI1 (CORONATINE INSENSITIVE 1) [8]. COI1 forms the SCF^COI1^ E3 ligase along with ARABIDOPSIS SKP-LIKE1 (ASK1)/ASK2, Cullin and RBX1 [8, 9]. JA-ZIM domain proteins (JAZs) are repressors of the JA signaling pathway [10–12], and they interact with and repress diverse downstream factors [13, 14]. COI1 serves as the primary JA receptor [15], and interacts with JAZs to form the COI1-JAZ coreceptor complex that effectively perceives bioactive JA forms (e.g. JA-Ile) [16–18]. JA signals trigger degradation of JAZ repressors via the SCF^COI1^-26S proteosome pathway, and activate diverse JA responses [10, 11, 19].

Previous studies demonstrated that exogenous application of JA enhances resistance to RKNs in tomato [20]. Consistently, *spr2*, a tomato JA-deficient mutant, displayed a RKN-susceptible phenotype compared with wild type [21, 22]. Recent studies revealed that some factors affect JA-regulated defense against the RKN *M. incognita* in tomato. For instance, miR319 and the transcription factor TCP4 regulate resistance to *M. incognita* by affecting JA contents [23]. The bHLH-type JA signaling transcription factor SlMYC2 participates in crosstalk between JA, strigolactone (SL), and abscisic acid (ABA) to inhibit resistance to *M. incognita* [24]. SlCSN4 and SlCSN5 interact with SlJAZ2 to positively regulate defense against *M. incognita* [25]. Although JAs control tomato defense against RKNs, the underlying molecular mechanism has not been fully explored, and remains to be elucidated.

WRKY transcription factors modulate plant defense against abiotic and biotic stresses [26–28]. The WRKY family in tomato contains 83 members [29]. SlWRKY70 confers resistance to aphids and the RKN *Meloidogyne javanica*, and its transcript level is inducible by salicylic acid (SA) but suppressed by JA [30]. Overexpression of *SlWRKY3* enhances resistance to the RKN *M. javanica*, whereas loss of function of *SlWRKY3* causes susceptibility [31]. SlWRKY45 represses tomato resistance to *M. javanica*, regarding the larger numbers of galls and females in roots with *Agrobacterium rhizogenes*-mediated *SlWRKY45*-overexpression [32]. Although SlWRKYs control tomato defense against RKNs, the regulatory mechanism is still unclear.

Here, we provided deep insights into the molecular mechanism by which SlWRKY45 negatively regulated defense against the RKN *M. incognita*. SlWRKY45 physically interacted with most SlJAZ members (SlJAZ1, SlJAZ2, SlJAZ3, SlJAZ4, SlJAZ5, SlJAZ6, SlJAZ7, and SlJAZ11). Loss of function of *SlWRKY45* enhanced resistance to the RKN *M. incognita*, whereas overexpression of *SlWRKY45* decreased defense against *M. incognita*. Furthermore, *SlWRKY45* overexpression reduced the contents of JA and JA-Ile under *M. incognita* infection, whereas SlWRKY45 bound to the promoter of the JA biosynthesis gene *ALLENE OXIDE CYCLASE* (*AOC*) and repressed its expression. Our results provide evidence that SlWRKY45 participates in both JA signaling and biosynthesis pathways, and inhibits resistance to *M. incognita* in tomato.

## Results

### SlJAZs interact with SlWRKY45

**Figure 1:**
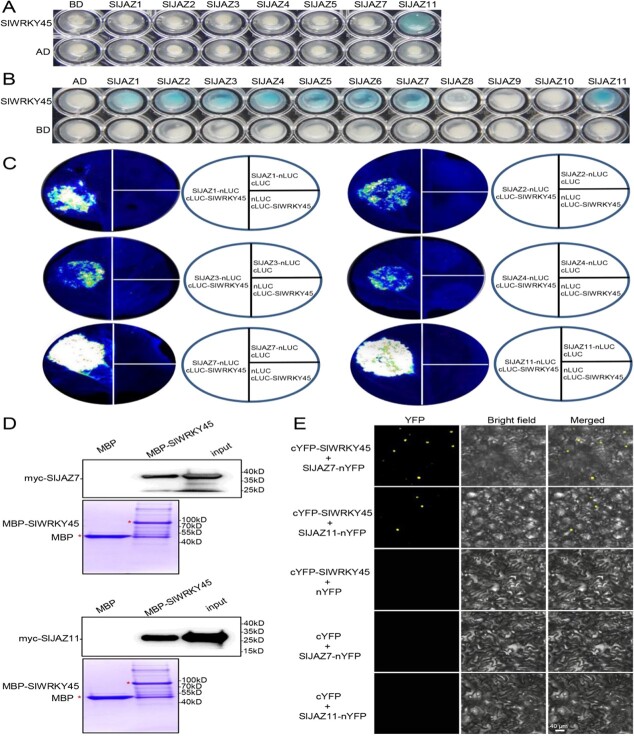
SlJAZ proteins interact with SlWRKY45. **A**–**B** Yeast two-hybrid (Y2H) assays to assess interactions of SlJAZs with SlWRKY45. SlJAZs and SlWRKY45 were fused to BD domain in pLexA (marked as BD-SlJAZs or BD-SlWRKY45) or B42AD domain in pB42AD (marked as AD-SlJAZs or AD-SlWRKY45). **C** Interactions of SlJAZs and SlWRKY45 were detected by firefly luciferase (LUC) complementation imaging (LCI) assays. SlJAZ1, SlJAZ2, SlJAZ3, SlJAZ4, SlJAZ7, SlJAZ11, and SlWRKY45 were fused with nLUC or cLUC (N or C-terminal fragments of LUC) to produce SlJAZ1-nLUC, SlJAZ2-nLUC, SlJAZ3-nLUC, SlJAZ4-nLUC, SlJAZ7-nLUC, SlJAZ11-nLUC, and cLUC-SlWRKY45, respectively. Luciferase activities were evaluated at 50 h after the infiltration of corresponding *Agrobacterium* strains in leaves of *N. benthamiana*. **D** Pull-down assays show that SlJAZ7 and SlJAZ11 associate with SlWRKY45. MBP and MBP-SlWRKY45 were immobilized on amylose resin, and incubated with transiently expressed myc-SlJAZ7 and myc-SlJAZ11 proteins. The samples were detected with an anti-myc antibody. **E** Bimolecular fluorescence complementation (BiFC) analyses show interaction of SlJAZ7/11 and SlWRKY45. SlJAZ7, SlJAZ11, and SlWRKY45 were fused with nYFP or cYFP (N or C-terminal parts of YFP), respectively. YFP signals were observed at 50 h after the expression of corresponding combinations of *Agrobacterium* strains in leaves of *N. benthamiana*.

To explore the molecular basis of JA pathway in regulation of tomato defense against *M. incognita*, we sought to identify potential downstream transcription factors of SlJAZ repressors using the yeast two-hybrid (Y2H) system. We first analysed expression levels of *SlJAZs* at 1 d, 3 d, 7 d, and 14 d after *M. incognita* infection, and found that the *SlJAZ11* expression was notably increased at these time points (Fig. S1, see online supplementary material). SlJAZ11 was selected as a bait and ligated with DNA binding domain (BD) in pLexA to screen a cDNA library of RKN-infected tomato. The WRKY transcription factor SlWRKY45 was identified as a candidate SlJAZ11-interaction protein (Fig. 1A). We further investigated whether other SlJAZs interacted with SlWRKY45 using Y2H assays. As shown in Fig. 1A, only BD-fused SlJAZ11 interacted with B42 activation domain (AD)-fused SlWRKY45 in yeast. We further fused SlJAZs with AD to produce AD-SlJAZs, and fused SlWRKY45 with BD to produce BD-SlWRKY45. As depicted in Fig. 1B, AD-SlJAZ1, SlJAZ2, SlJAZ3, SlJAZ4, SlJAZ5, SlJAZ6, SlJAZ7, and SlJAZ11 interacted with BD-SlWRKY45 in yeast, whereas AD-SlJAZ8, SlJAZ9, and SlJAZ10 did not.

Firefly luciferase (LUC) complementation imaging (LCI) assays in Figs. 1C and S2A (see online supplementary material) showed that coexpression of nLUC (N-terminal fragment of LUC)-fused SlJAZ1, SlJAZ2, SlJAZ3, SlJAZ4, SlJAZ7, or SlJAZ11 (SlJAZ1-nLUC, SlJAZ2-nLUC, SlJAZ3-nLUC, SlJAZ4-nLUC, SlJAZ7-nLUC, SlJAZ11-nLUC), and cLUC (C-terminal fragment of LUC)-fused SlWRKY45 reconstituted LUC activity in *Nicotiana benthamiana* leaves, while coexpression of SlJAZ10-nLUC/cLUC-SlWRKY45 and the negative controls did not. Furthermore, pull-down assays exhibited that MBP-fused SlWRKY45 pulled down transiently expressed myc-fused SlJAZ7 and myc-fused SlJAZ11, but MBP could not (Fig. 1D). Meanwhile, MBP-fused SlWRKY45 was unable to pull down myc-fused SlJAZ10 (Fig. S2B, see online supplementary material).

Bimolecular fluorescence complementation (BiFC) assays showed that YFP signals were detected in the nucleus of epidermal *N. benthamiana* cells when SlJAZ7 or SlJAZ11 in fusion with N-terminal part of YFP (SlJAZ7-nYFP or SlJAZ11-nYFP) was coexpressed with cYFP (C-terminal part of YFP)-fused SlWRKY45 (cYFP-SlWRKY45), while YFP signals could not be detected when SlJAZ10-nYFP and cYFP-SlWRK45, or the negative control combinations were coexpressed (Fig. S2C, see online supplementary material). The results of BiFC assays suggest that SlJAZ7 and SlJAZ11 interact with SlWRKY45 in the nucleus.

**Figure 2:**
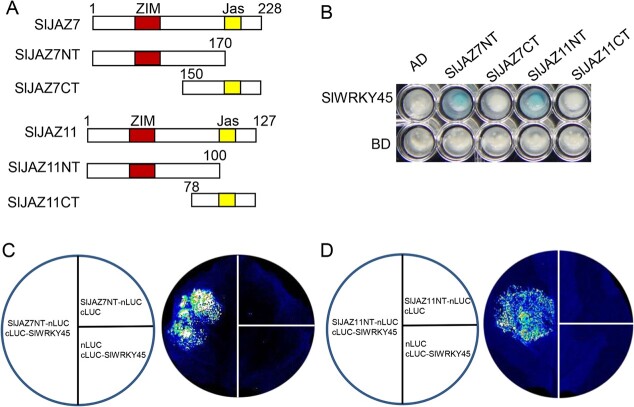
The N-terminal parts of SlJAZs mediate interaction between SlJAZs and SlWRKY45. **A** Diagrams of SlJAZ7 and SlJAZ11 domain constructs. Red and yellow boxes represent the ZIM and Jas domains, respectively. **B** Y2H assays showing interactions of SlJAZ7 and SlJAZ11 domain constructs with SlWRKY45. The corresponding domains of SlJAZ7 and SlJAZ11, and SlWRKY45 were fused with AD or BD domains, respectively. **C**–**D** Interactions of the N-terminal parts of SlJAZ7 (**C**) and SlJAZ11 (**D**) with SlWRKY45 were analysed by LCI assays. N-terminal fragments of SlJAZ7 and SlJAZ11, and SlWRKY45 were in fusion with nLUC and cLUC, respectively. Luciferase activities were determined at 50 h after the injection of corresponding *Agrobacterium* strains in leaves of *N. benthamiana*.

SlJAZ7 and SlJAZ11 were truncated to generate N and C-terminal fragments (SlJAZ7NT, SlJAZ11NT, SlJAZ7CT, and SlJAZ11CT), containing the ZIM or Jas domain, respectively (Fig. 2A), in order to investigate which domains of SlJAZs mediate interaction with SlJAZs and SlWRKY45. As illustrated in Fig. 2B, SlWRKY45 interacted with SlJAZ7NT and SlJAZ11NT, but not with SlJAZ7CT and SlJAZ11CT. LCI assays further showed that co-infiltration of SlJAZ7NT-nLUC or SlJAZ11NT-nLUC with cLUC-SlWRKY45 reconstituted strong LUC signals (Figs. 2C and D). These results consistently confirm that the N-terminal parts of SlJAZs participate in SlJAZ-SlWRKY45 interactions.

Taken together (Figs. 1 and 2), these results consistently reveal that SlWRKY45 interacts with SlJAZs.

### Nuclear localization and *M. incognita* infection-induced expression of SlWRKY45

**Figure 3:**
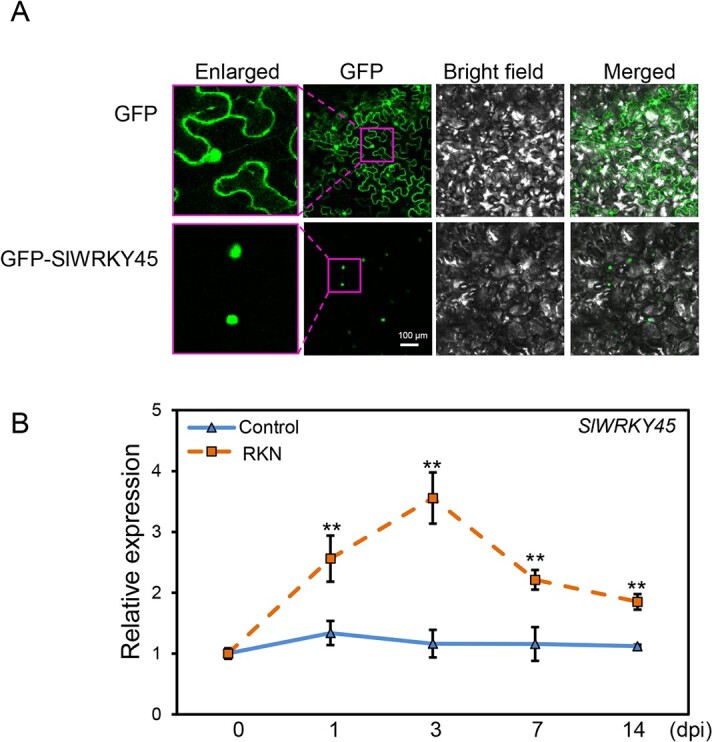
SlWRKY45 is localized to the nucleus and is inducible by the RKN *Meloidogyne incognita* infection. **A** SlWRKY45 is localized to the nucleus. *Agrobacterium* strains GV3101 carrying the empty vector or GFP-fused SlWRKY45 vector were injected into leaves of *N. benthamiana* to transiently express GFP and GFP-fused SlWRKY45 protein*.* GFP signals were detected using a confocal microscope at 50 h after injection. **B** Relative expression level of *SlWRKY45* in the roots of CM wild type (WT) at the indicated time points after infection without (control) or with *Meloidogyne incognita*. Values represent means (±SD) of three independent biological replicates. Significant differences between *Meloidogyne incognita*-infected roots of CM and the corresponding control were analysed by Student’s *t* test (^**^P < 0.01) and indicated with asterisks.

We further ligated SlWRKY45 to the C-terminus of GFP (GFP-SlWRKY45) to analyse its subcellular localization. In contrast to the localization of GFP alone, GFP-SlWRKY45 protein signals were detected in the nucleus, suggesting that SlWRKY45 is localized to the nucleus (Fig. 3A). Consistent with the interaction of SlJAZ7/11 and SlWRKY45 in the nucleus, SlJAZ7-GFP and SlJAZ11-GFP were also localized to the nucleus (Fig. S3, see online supplementary material).

In addition, we explored the expression pattern of *SlWRKY45*, and discovered that the expression levels of *SlWRKY45* in roots were strongly increased at 1 d, 3 d, 7 d, and 14 d after the RKN *M. incognita* infection, with a peak at 3 d (Fig. 3B), implying that SlWRKY45 may be involved in defense against *M. incognita*.

### Loss of *SlWRKY45* function enhances tomato defense against *M. incognita*

To investigate the function of *SlWRKY45*, we generated *slwrky45* mutants in tomato (*Solanum lycopersicum*) cv Castlemart (CM) using CRISPR/Cas9 technology. Two targets specific for the first and second exons of *SlWRKY45*, respectively, were selected and ligated with the guide RNA scaffold in the CRISPR/Cas9 vector pCBSG012 (Fig. S4 , see online supplementary material). Thirteen independent T0 transgenic lines (*slwrky45-cr-1* to *slwrky45-cr-13*) were obtained through *Agrobacterium*-mediated transformation (Fig. S5A, see online supplementary material). Sequencing-based genotyping showed that the *slwrky45* mutations were homozygous in *slwrky45-cr-1* and *slwrky45-cr-2*, heterozygous in *slwrky45-cr-10* and *slwrky45-cr-13*, bi-allelic in *slwrky45-cr-3*, *slwrky45-cr-9* and *slwrky45-cr-11*, and chimeric in *slwrky45-cr-5* and *slwrky45-cr-7*, and it was wild type (WT) in the remaining four lines (Fig. S5A, see online supplementary material). The editing rate was 69.2% (Fig. S5B, see online supplementary material).

The two representative T0 homozygotes, *slwrky45-cr-1* (16-bp and 1-bp deletions, respectively, in the first and second target) and *slwrky45-cr-2* (3-bp and 1-bp deletions, respectively, in the first and second target) were chosen for further study (Fig. S5A, see online supplementary material). We first analysed the presence and absence of the *slwrky45* mutations and *Cas9* in 15 T1 generation plants of *slwrky45-cr-1* and *slwrky45-cr-2*, respectively. As shown in Fig. S6 (see online supplementary material), all the detected T1 generation contained the same *slwrky45* mutations as their corresponding parental T0 lines, suggesting that these mutations were stably inherited by their T1 progeny. Meanwhile, *Cas9*-free T1 homozygotes were identified (three plants from *slwrky45-cr-1* and four plants from *slwrky45-cr-2*). Furthermore, we investigated the three most probable off-target sites of the two single guide RNAs in the *slwrky45-cr-1* and *slwrky45-cr-2* T1 plants, and found that no mutations occurred at these off-target sites (Fig. S7 and Table S1, see online supplementary material). Therefore, *Cas9*-free T2 homozygotes of *slwrky45-cr-1* and *slwrky45-cr-2* (Fig. S8, see online supplementary material) were used for ensuing studies.

**Figure 4:**
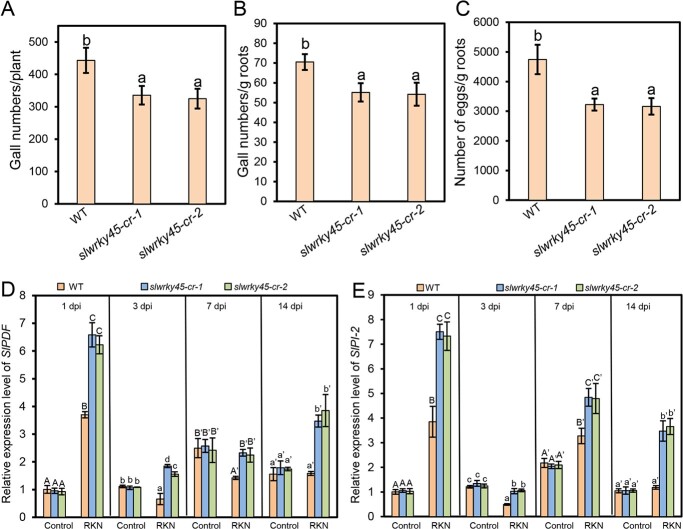
The *slwrky45* mutant exhibits increased resistance to the RKN *Meloidogyne incognita.***A**–**C** Gall numbers per plant (**A**), gall numbers per gram of roots (**B**), and numbers of eggs per gram of roots (**C**) in the CM wild type and *slwrky45* mutants (*slwrky45-cr-1* and *slwrky45-cr-2*) at 35 d after *M. incognita* infection. Data represent means (±SD) of 20 plants. Significant differences were analysed by ANOVA with Duncan’s multiple range test (*P* < 0.05) and indicated with letters. **D**–**E** Relative expression levels of *SlPDF* (**D**) and *SlPI-2* (**E**) at 1 d, 3 d, 7 d, and 14 d post infection without (control) or with *M. incognita* from the roots of the indicated plants. Data represent means (±SD) of three independent biological replicates. Significant differences were analysed by ANOVA with Duncan’s multiple range test (*P* < 0.05) and indicated with letters.

The CM wild type, *slwrky45-cr-1*, and *slwrky45-cr-2* plants were inoculated with *M. incognita*. At 7 d and 35 d after inoculation, the number of galls for each root were counted. The results in Fig. S9A and B (see online supplementary material) showed that, at 7 d post inoculation, the gall numbers per gram of roots were significantly lower in *slwrky45-cr-1* and *slwrky45-cr-2* compared with those in WT. Furthermore, we found that at 35 d post inoculation, the gall numbers per plant, and gall numbers and egg numbers per gram of roots in *slwrky45-cr* mutants were also significantly lower compared with those in WT (Fig. 4A–C), consistently indicating that SlWRKY45 plays a negative role in defending against *M. incognita*. *PLANT DEFENSE FACTOR* (*PDF*) and *PROTEINASE INHIBITOR 2* (*PI-2*) are two defensive genes against pathogens, including *M. incognita* [24]. *SlPDF* and *SlPI-2* expression was responsive to *M. incognita* infection as shown in Fig. 4D and E. Consistent with the increased resistance of *slwrky45-cr* plants, the expression levels of *SlPDF* and *SlPI-2* were obviously higher in *slwrky45-cr* roots with the RKN *M. incognita* infection compared with those in the *M. incognita*-infected WT roots (Fig. 4D and E).

### Overexpression of *SlWRKY45* reduces resistance to *M. incognita* in tomato

**Figure 5:**
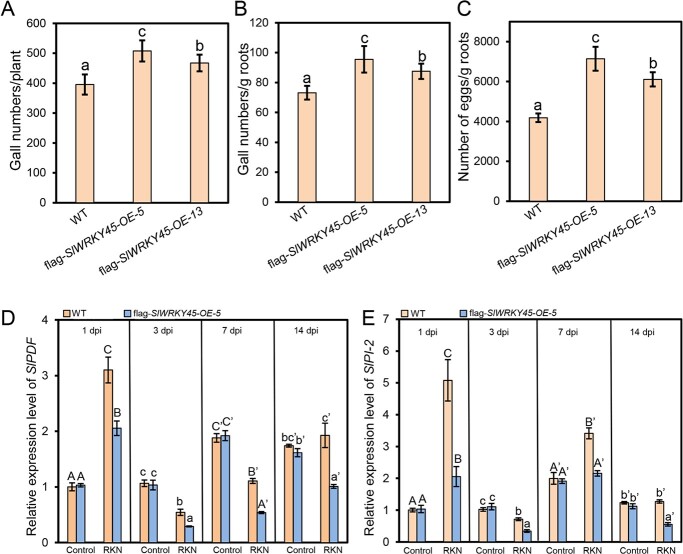
Overexpression of *SlWRKY45* represses resistance to the RKN *Meloidogyne incognita* in tomato. **A**–**C** Gall numbers per plant (**A**), gall numbers per gram of roots (**B**), and numbers of eggs per gram of roots (**C**) in the CM wild type and *SlWRKY45*-overexpressing plants (flag-*SlWRKY45-OE-5* and flag-*SlWRKY45-OE-13*) at 35 d after *M. incognita* infection. Data represent means (±SD) of 20 plants. Significant differences were analysed by ANOVA with Duncan’s multiple range test (*P* < 0.05) and indicated with letters. **D**–**E** Relative expression levels of *SlPDF* (**D**) and *SlPI-2* (**E**) in roots of the indicated plants at 1 d, 3 d, 7 d, and 14 d after infection without (control) or with *M. incognita*. Data represent means (±SD) of three independent biological replicates. Significant differences were analysed by ANOVA with Duncan’s multiple range test (*P* < 0.05) and indicated with letters.

We further generated *SlWRKY45*-overexpressing lines of tomato. Flag-*SlWRKY45-OE-5* and flag-*SlWRKY45-OE-13* with approximately 39-fold and 28-fold of the WT level regarding *SlWRKY45* expression, respectively, were used as representatives (Fig. S9C, see online supplementary material). The CM wild type and flag-*SlWRKY45-OE* plants were inoculated with *M. incognita*. The gall numbers per plant in flag-*SlWRKY45-OE* roots at 7 d after infection, and the gall numbers per plant, gall numbers and egg numbers per gram of roots in flag-*SlWRKY45-OE* plants at 35 d after infection were larger than those in the infected WT roots at 7 d, and 35 d, respectively (Figs S9D (see online supplementary material) and 5A–C). We further analysed the transcript levels of *SlPDF* and *SlPI-2* in the roots of WT and flag-*SlWRKY45-OE* lines with *M. incognita* infection. As shown in Fig. 5D and E, the expression of *SlPDF* and *SlPI-2* in the roots of flag-*SlWRKY45-OE* lines at 1 d, 3 d, 7 d, and 14 d after infection was significantly lower than those in the infected WT roots.

Altogether, the results in Figs. 4, 5, and S9 (see online supplementary material) demonstrate that SlWRKY45 acts as a repressor to regulate resistance to *M. incognita* in tomato.

### *SlWRKY45* overexpression attenuates RKN-affected JAs biosynthesis in tomato

**Figure 6:**
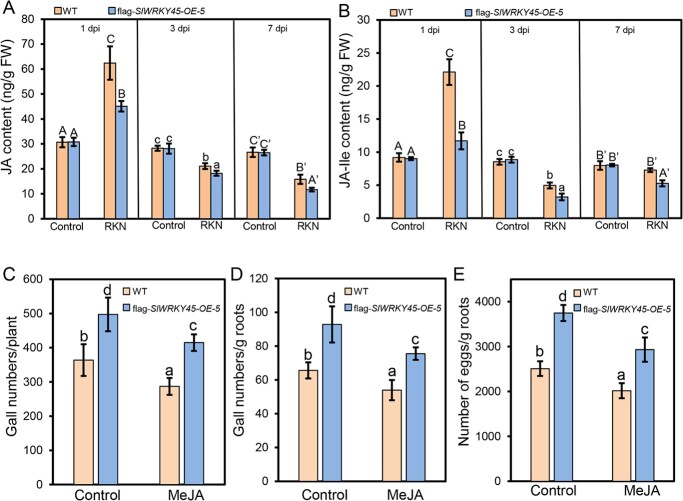
Overexpression of *SlWRKY45* inhibits RKN-regulated JA biosynthesis in tomato. **A**–**B** Contents of jasmonic acid (JA) (**A**) and JA-isoleucine (JA-Ile) (**B**) in roots of the CM wild type and *SlWRKY45*-overexpressing plants (flag-*SlWRKY45-OE-5*) at 1 d, 3 d, and 7 d after inoculation without (control) or with *Meloidogyne incognita*. Data represent means (±SD) of three independent biological replicates. Significant differences were analysed by ANOVA with Duncan’s multiple range test (*P* < 0.05) and indicated with letters. **C**–**E** Gall numbers per plant (**C**), gall numbers per gram of roots (**D**), and numbers of eggs per gram of roots (**E**) in the CM wild type and the *SlWRKY45*-overexpressing plants flag-*SlWRKY45-OE-5* without (control) or with MeJA treatment. The plants were inoculated with *M. incognita* at 24 h after being treated without (control) or with 100 μM MeJA, and the resistance to *M. incognita* was assessed at 35 d after infection. Data represent means (±SD) of 20 plants. Significant differences were analysed by ANOVA with Duncan’s multiple range test (*P* < 0.05) and indicated with letters.

To further explore the mechanism of SlWRKY45 in regulating the resistance to *M. incognita*, we examined the concentrations of JA and JA-Ile in the roots of WT and *SlWRKY45-*overexpressing plants (flag-*SlWRKY45-OE-5* as a representative) without or with *M. incognita* inoculation. In contrast to those in WT roots without *M. incognita* infection, the contents of JA and JA-Ile in *M. incognita*-infected roots of WT were increased at 1 d after infection, and were restored to normal levels or even decreased at 3 d and 7 d (Fig. 6A and B), which were consistent with previous observations [33]. The contents of JA and JA-Ile in the roots of flag-*SlWRKY45-OE-5* at 1 d, 3 d, and 7 d after *M. incognita* infection were lower compared with those in the roots of *M. incognita*-infected WT (Fig. 6A and B).

In addition, the CM wild type and flag-*SlWRKY45-OE-5* plants were treated with MeJA, and 24 h later, these plants were infected with *M. incognita* for 7 d and 35 d. The results in Figs. S10 (see online supplementary material) and 6C–E showed that MeJA treatment rescued the resistance of flag-*SlWRKY45-OE-5* plants to *M. incognita*. These results indicate that *SlWRKY45-*overexpression reduces resistance to *M. incognita* partially by decreasing JAs concentration.

### *SlWRKY45* binds to and represses the JA biosynthesis gene *SlAOC*

**Figure 7:**
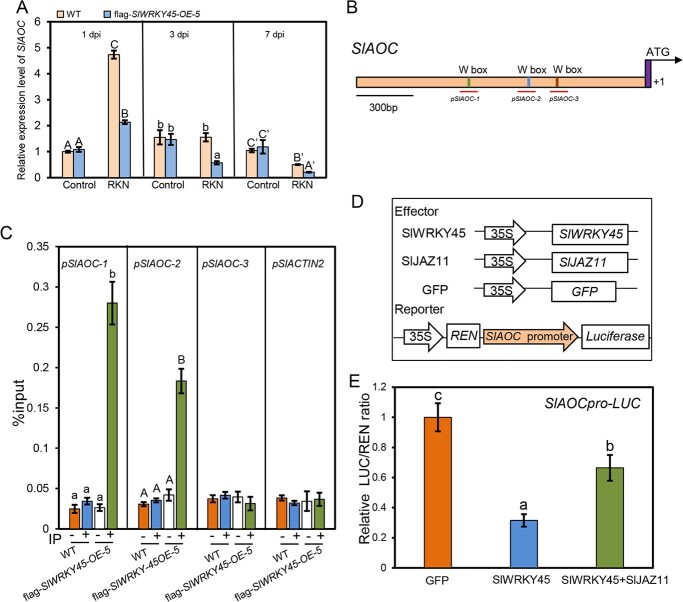
SlWRKY45 binds and represses the expression of *SlAOC*. **A** Relative expression level of *SlAOC* at 1 d, 3 d, and 7 d post infection without (control) or with *Meloidogyne incognita* in the indicated plants. Data represent means (±SD) of three independent biological replicates. Significant differences were analysed by ANOVA with Duncan’s multiple range test (*P* < 0.05) and indicated with letters. **B** Diagram of *SlAOC* promoter. Red lines represent the regions detected in ChIP-qPCR assays. **C** ChIP-qPCR assays to detect the binding of flag-SlWRKY45 to *SlAOC* promoter. Chromatin from RKN-infected CM wild type and the flag-*SlWRKY45* transgenic plants was immunoprecipitated without (−) or with (+) anti-flag antibody. A promoter of *SlACTIN2* was used as a negative control. Data represent means (±SD) of three independent biological replicates. Significant differences were analysed by ANOVA with Duncan’s multiple range test (*P* < 0.05) and indicated with different letters. **D** Diagram displaying the constructs used in the Dual-LUC assays in (**E**). **E** Dual-LUC assays showing that SlWRKY45 attenuates *SlAOC* promoter activity, and that SlJAZ11 inhibits this effect. Error bars represent SD (*n* = 6). Significant differences were analysed by ANOVA with Duncan’s multiple range test (*P* < 0.05) and indicated with letters.

A previous study showed that two JA biosynthesis genes *LIPOXYGENASE D* (*LOXD*) and *ALLENE OXIDE CYCLASE* (*AOC*) were induced by the RKN *M. incognita* infection in tomato [25]. Thus, we explored whether the decreased JAs contents in *M. incognita*-infected *SlWRKY45-*overexpressing plants were due to reduced expression of *SlLOXD* or *SlAOC* under *M. incognita* infection. To demonstrate this, we analysed the transcript levels of *SlLOXD* and *SlAOC*, and discovered that, in response to *M. incognita* infection, the expression levels of *SlAOC* were lower in *SlWRKY45-*overexpression lines compared with those in WT (Fig. 7A), while *SlLOXD* expression was not affected (Fig. S11 , see online supplementary material).

We next investigated whether SlWRKY45 was able to bind to and regulate *SlAOC*. We carried out chromatin immunoprecipitation-quantitative PCR (ChIP-qPCR) analysis using *M. incognita*-infected flag-*SlWRKY45-OE-5* transgenic plants, and found that SlWRKY45 bound to the first and second typical WRKY factor target sequences (W-box, TTGACT) in the promoter of *SlAOC* (Fig. 7B and C). We further examined the regulatory function of SlWRKY45 on the *SlAOC* promoter using Dual-LUC assays in which the *SlAOC* promoter drove the *luciferase* (*LUC*) reporter gene (*SlAOC*_pro_-LUC), and *SlWRKY45* controlled by the CaMV35S promoter (35S-*SlWRKY45*) served as an effector (Fig. 7D). As shown in Fig. 7E, coexpression of *SlAOC*_pro_-LUC and 35S-*SlWRKY45* in *N. benthamiana* leaves resulted in a lower LUC/REN ratio than the coexpression of *SlAOC*_pro_-LUC and the control 35S-*GFP*, consistently suggesting that SlWRKY45 obviously represses the promoter and expression of *SlAOC*. Moreover, we found that the repression activity of SlWRKY45 was attenuated by SlJAZ11 (Fig. 7E), suggesting that SlJAZ11 inhibits the function of SlWRKY45.

## Discussion

Previous studies have indicated that WRKY transcription factors associate with JA pathway to exert their biological functions. For example, AtWRKY57 integrates both auxin and JA signaling by interacting with AtIAA29 and AtJAZs, and mediates leaf senescence [34]. AtWRKY51 associates with AtJAZ8/AtJAV1 to comprise a JAV1-JAZ8-WRKY51 complex for controlling defense against insects [35]. SlWRKY31 cooperates with SlVQ15, and participates in JA signaling and resistance to *Botrytis cinerea* [36]. Nevertheless, the relationship between JA and WRKYs in tomato remains poorly understood. Here, we reveal that SlWRKY45 is involved in both JA biosynthesis and signaling pathways to attenuate resistance to the RKN *M. incognita* (Figs 1–7).

WRKYs play regulatory roles via interacting with various proteins. AtWRKY50 interacts with AtTGA2 or AtTGA5 to form a protein complex and synergistically activates the expression of the resistance-related gene *PATHOGENESIS-RELATED* 1 (*PR1*) [37]. AtWRKY8 recruits AtVQ10 to enhance its binding to target DNA, and promote resistance to *B. cinerea* [38]. AtVQ9 associates with and decreases the transcriptional activity of AtWRKY8, and modulates tolerance to salt stress [39]. AtWRKY12 and AtWRKY13 interact with SQUAMOSA PROMOTER BINDING-LIKE 10 to antagonistically regulate their transcriptional functions and age-mediated flowering [40]. AtWRKY38 and AtWRKY62 associate with HISTONE DEACETYLASE 19 to participate in defense responses [41]. Here, we demonstrate that SlJAZs act through their N-terminal regions to physically interact with SlWRKY45 using Y2H, LCI, pull-down, and BiFC assays (Figs. 1 and 2).

WRKYs bind to target genes and their own promoters to activate or repress expression through the combination of the WRKY domain and W-box. For instance, AtWRKY57 binds to the W-box region of the *AtJAZ1* and *AtJAZ5* promoters and activates their expression to repress JA-mediated defense against *B. cinerea* [42]. AtWRKY51 binds to the promoter regions of the Arabidopsis JA biosynthesis gene *ALLENE OXIDE SYNTHASE* (*AOS*) via the W-box sequence [35]. It also associates with AtJAV1 and AtJAZ8 to repress *AtAOS* expression, which inhibits JAs biosynthesis and controls resistance to insect attack [35]. PcWRKY1 binds to the W-boxes in its own promoter, as well as the *PcWRKY3* and *PcPR1–1* promoters [43]. Here, our ChIP-qPCR results showed that SlWRKY45 binds to fragments spanning the W-boxes in the promoter of the JA biosynthesis gene *SlAOC* (Fig. 7C). Dual-LUC assays suggested that SlWRKY45 attenuates the expression of *SlAOC* (Fig. 7E). It would be interesting to further investigate whether SlWRKY45 could bind to its own promoter and regulate its own expression.

One previous study reported that *SlWRKY45* expression is enhanced within 5 d after *M. javanica* infection, and is maintained through feeding-site development and gall formation [32]. The phytohormones cytokinin (CK), auxin, and SA induce *SlWRKY45* expression, whereas JA inhibits its expression. In response to the RKN *M. javanica* infection, the roots with transient *SlWRKY45* overexpression displayed higher numbers of developed females, gall formation, and overall feeding site area than WT roots. In this study, we found that SlWRKY45 was localized to the nucleus, and was induced at 1 d, 3 d, 7 d, and 14 d after *M. incognita* infection (Fig. 3). We generated stable *SlWRKY45*-overexpressing transgenic tomato and *slwrky45* mutants, and demonstrated that SlWRKY45 is a negative regulator of tomato defense against *M. incognita* (Figs. 4, 5, and S9, see online supplementary material). Moreover, we deeply explored and revealed the mechanism that SlWRKY45 interacts with SlJAZs, directly binds to the *SlAOC* promoter, and inhibits *SlAOC* expression and JA biosynthesis to reduce defense against the RKN *M. incognita* (Figs. 1, 2, 6, and 7).

SlMYC2, a master transcription factor, interacts with 11 SlJAZs [44], and plays positive or negative roles to modulate diverse physiological responses in tomato, including positively regulating fruit chilling tolerance [45], resistance to the necrotrophic pathogen *B. cinerea* [44, 46], growth and developmental processes such as flower formation, fruit set, and fruit shape [47], and negatively controlling resistance to *M. incognita* by mediating the interplay of SL, ABA, and JA [24]. In our results, we discovered that SlWRKY45 interacts with most SlJAZ proteins, and negatively controls defense against *M. incognita* (Figs. 1, 4, 5, and S9, see online supplementary material). It remains to explore whether SlWRKY45 also controls other JA-regulated responses in tomato. As shown by the finding that the transcription factors SlMYC2 and SlWRKY45 both repress resistance to *M. incognita* in tomato [24] (Figs. 4, 5, and S9, see online supplementary material), it remains to investigate whether SlMYC2 and SlWRKY45 target some mutual downstream genes to synergistically or antagonistically regulate their expression, and control defense responses. Additionally, it will be interesting to isolate the master factors positively modulating JA-mediated tomato defense against RKNs, which will contribute to further understanding the molecular basis of JA-controlled defense responses.

**Figure 8:**
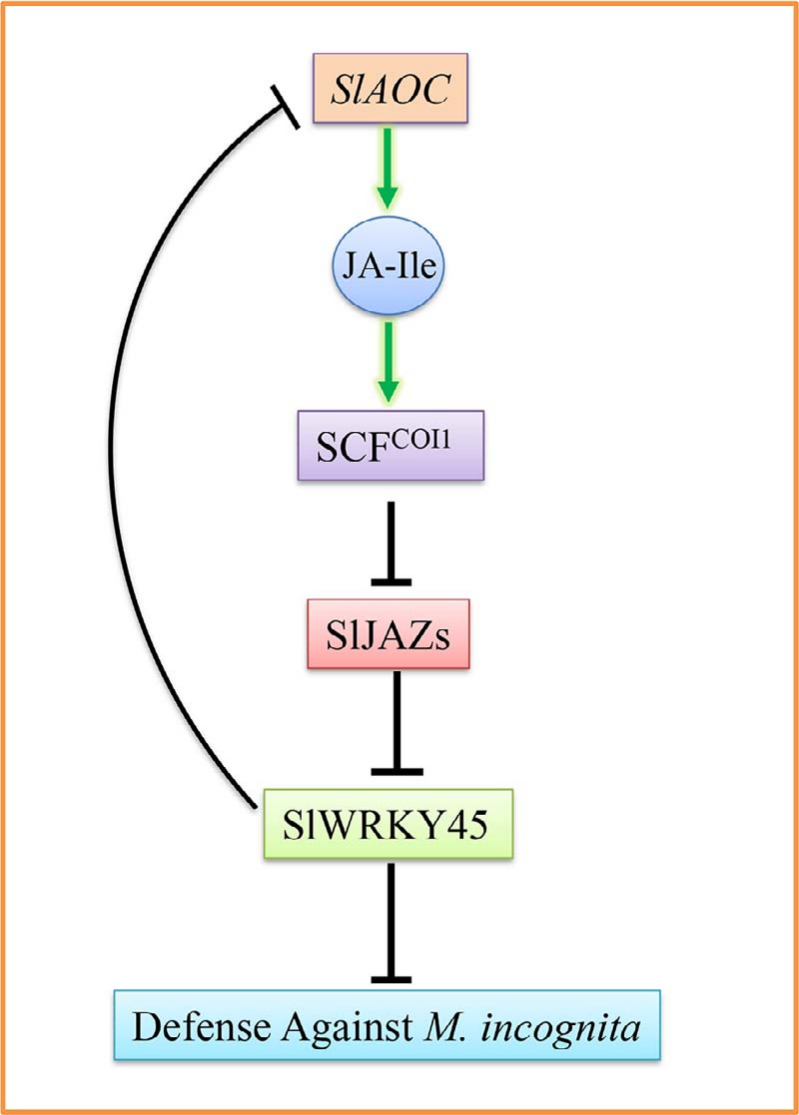
A simplified model of SlWRKY45 in JA-mediated defense against the RKN *Meloidogyne incognita* in tomato. SlJAZs interact with SlWRKY45 to repress the regulation of downstream genes. JA-Ile triggers degradation of SlJAZs through the SCF^COI1^ complex to release SlWRKY45. The released SlWRKY45 represses *SlAOC* expression, inhibits RKN-affected JAs biosynthesis, and attenuates tomato resistance to *M. incognita*.

A summary of our findings is shown in Fig. 8. SlJAZ repressors interact with and repress SlWRKY45 to attenuate its function. JAs induce SlJAZs degradation to release SlWRKY45. The released SlWRKY45 binds to and inhibits *SlAOC* expression to reduce RKN-regulated JAs biosynthesis, and represses defense against the RKN *M. incognita*.

## Materials and methods

### Plant materials and growth conditions

Seeds of CM wild type, *SlWRKY45*-overexpressing plants and *slwrky45* mutants were germinated at 28°C on moistened filter paper for 2–3 days, and then grown in a greenhouse (24°C–26°C/16°C–18°C,16 h light/8 h dark). Seeds of *N. benthamiana* were sown in soil, and grown in a greenhouse (25°C–28°C/16°C–18°C,16 h light/8 h dark).

### Generation of *SlWRKY45*-overexpression plants

The CDS of *SlWRKY45* was amplified with specific primers (Table S2, see online supplementary material), and ligated into a reformative pCAMBIA1300 vector through the *Sal* I and *Spe* I sites to generate the *SlWRKY45*-overexpression plasmid (flag-*SlWRKY45-OE*), in which *SlWRKY45* was fused with three flag tags and was driven by the *CaMV35S* promoter. Through *Agrobacterium* (GV3101)-mediated cotyledon explant transformation, this construct was transformed into CM wild type. Hygromycin B was used to select the transgenic lines. T3 homozygous *SlWRKY45*-overexpressing plants were used for further experiments.

### Generation of *SlWRKY45* gene-edited plants

CRISPR/Cas9 technology was used to generate *slwrky45* mutants. We used CRISPR-P to choose two sgRNAs that targeted the first and second exons of *SlWRKY45* (Fig. S4A, see online supplementary material). The PCR primers included the two target sites and the *Bsa* I site, which are shown in Table S2, see online supplementary material. The PCR fragment was amplified using the plasmid pSG-SlU6 (Biogle GeneTech, Jiangsu, China) as a template to generate the PCR product containing the *Bsa* I site, two targets, gRNA scaffold, and the tomato U6 promoter (Fig. S4B, see online supplementary material). The product was purified and inserted into the pCBSG012 vector (Biogle GeneTech, Jiangsu, China) through the *Bsa* I site (Fig. S4C–D, see online supplementary material). Through *Agrobacterium* (GV3101)-mediated cotyledon explant transformation, this construct was transformed into CM wild type. Hygromycin B was used to select the transformants. *slwrky45* mutants of *Cas9*-free T2 homozygotes were analysed by PCR and sequencing, and used for further experiments.

### Analysis of mutation types and off-target mutations

Tomato DNA was extracted using a DNA extraction kit (GeneBette, Beijing, China), and used as a template to amplify the target sites using PrimeSTAR Max DNA polymerase (TaKaRa, Ohtsu, Japan). To analyse mutation types of each T0 line, we cloned the PCR fragments into the pMD20-T vector (TaKaRa, Ohtsu, Japan), sent 15 individual clones for sequencing, and analysed the mutations. To analyse mutation types of T1 and T2 lines, the PCR products were sequenced. The primers used for amplification are shown in Table S3, see online supplementary material. To identify *Cas9*, the PCR products were amplified using primers specific for *Cas9* (Table S4, see online supplementary material).

To analyse off-target mutations, we used CRISPR-P to predict the potential off-target sites (Table S1, see online supplementary material). The corresponding primers (Table S5, see online supplementary material) for each site were used for PCR amplification using PrimeSTAR Max DNA polymerase (TaKaRa, Ohtsu, Japan), and the PCR products were sequenced.

### Yeast two-hybrid screening and yeast two-hybrid assays

SlJAZ11 was ligated to the pLexA vector with BD, and the cDNA library generated with RKN-infected tomato was used for yeast two-hybrid (Y2H) screening. Y2H screening was carried out based on the manufacturer’s instructions (Clontech, CA, USA). For Y2H assays, full-length CDSs or fragments of SlJAZ1, SlJAZ2, SlJAZ3, SlJAZ4, SlJAZ5, SlJAZ6, SlJAZ7, SlJAZ8, SlJAZ9, SlJAZ10, SlJAZ11, and SlWRKY45 were fused with the pLexA or pB42AD vector. Yeast transformation and analysis of protein interactions were carried out in line with the manufacturer’s instructions (Clontech, CA, USA). All Y2H experiments were repeated three biological times.

### LCI assays

The CDSs of SlJAZ1, SlJAZ2, SlJAZ3, SlJAZ4, SlJAZ7, SlJAZ10, SlJAZ11, SlJAZ7NT, SlJAZ11NT, and SlWRKY45 were amplified with primers listed in Table S2 (see online supplementary material), and cloned into the pCAMBIA-nLUC or pCAMBIA-cLUC vector, respectively [48]. *Agrobacterium* strains (GV3101) carrying the indicated vectors were cultured, suspended, mixed for the indicated recombinant pairs, stranded for 3–5 h and injected into the leaves of *N*. *benthamiana*. 50 h later, a luciferin solution (0.1 mM luciferin, 0.1% Tween 20, 1 mM NaOH) was sprayed onto the leaves. Photos were obtained by a Tanon 5200Multi instrument (Tanon, Shanghai, China). The LCI assays were repeated three biological times.

### BiFC assays

The CDSs of SlWRKY45, SlJAZ7, SlJAZ10, and SlJAZ11 were fused into the cYFP or nYFP vector [49]. *Agrobacterium* GV3101 carrying the indicated construct pairs was injected into leaves of *N. benthamiana*. At 50 h after injection, YFP signals were captured using a confocal microscope (TCS-SP5, Leica, Wetzlar, Germany). BiFC assays were repeated three biological times. The primers are shown in Table S2, see online supplementary material.

### Pull-down assays

The CDS region of SlWRKY45 was cloned to the pMAL-c5X vector (NEB, MA, USA) via the *Sal* I and *EcoR* I sites for MBP fusion. *Escherichia coli* strains Transetta (DE3) containing the MBP-fused SlWRKY45 vector or empty vector were cultured at 16°C overnight in LB liquid medium with 0.3 mM IPTG to induce the expression of the corresponding proteins. Amylose resin (NEB, MA, USA) was used to purify these proteins.

The SlJAZ7, SlJAZ10, and SlJAZ11 were inserted into the pROK2 vector via the *Sma* I and *Sac* I sites to generate the constructs myc-SlJAZ7, myc-SlJAZ10, and myc-SlJAZ11. These vectors were respectively transformed into *Agrobacterium* strain (GV3101), and infiltrated into leaves of *N. benthamiana*. At 50 h later, 3 g of the corresponding leaves transiently expressing myc-SlJAZ7, myc-SlJAZ10, or myc-SlJAZ11 were harvested to extract total proteins using RB buffer (25 mM imidazole, protease inhibitor cocktail, 100 mM NaCl, 20 mM 2-mercaptoethanol, 10% glycerol, 0.1% Tween 20, and 50 mM Tris–HCl, pH 7.8).

Pull-down assays were adopted using previously described methods [49] with slight modification. MBP-fused SlWRKY45 and MBP proteins were respectively added to the amylose resin for 4 h at 4°C, after which 200 μL myc-SlJAZ7, myc-SlJAZ10, or myc-SlJAZ11 protein was added and incubated for 2 h at 4°C. After washing four to six times, the samples were boiled with 100 μL SDS loading buffer at 95°C for 10 min. The proteins were separated via 10% SDS-PAGE for 2 h, and then transferred to PVDF membranes (Millipore, MA, USA). The PVDF membranes were blocked using PBS buffer with 5% nonfat milk, and subsequently incubated with an anti-myc antibody (Abmart, Shanghai, China; 1:5000 dilutions) for 1 h and a goat anti-mouse secondary antibody (Abmart, Shanghai, China; 1:3000 dilutions) for 1 h. The proteins were observed using enhanced chemiluminescence (ECL) (Solarbio, Beijing, China) by a Tanon 5200Multi instrument (Tanon, Shanghai, China). The pull-down assays were repeated three biological times.

### Quantitative real-time PCR

RNA isolation and cDNA synthesis were respectively conducted using kits (DP432, Tiangen, Beijing, China; and AT311–02, Transgen, Beijing, China) in line with the manufacturer’s instructions. qRT-PCR using SYBR Green Mix (TaKaRa, Ohtsu, Japan) with specific primers (Table S6, see online supplementary material) on the Bio-Rad CFX96 qPCR instrument was conducted to detect the expression levels of genes. The qRT-PCR reaction conditions were as below: 3 min at 95°C, 39 cycles of 15 s at 95°C, 10 s at 56°C, and 72°C for 15 s. The internal control gene was tomato *ACTIN2*. Values of relative gene expression were analysed using the 2^−ΔΔCt^ method [50]. The qRT-PCR experiments were repeated three biological times.

### Subcellular localization

SlWRKY45 was in fusion with GFP in the pEGAD vector through the *EcoR* I and *BamH* I sites to produce the GFP-SlWRKY45 construct. SlJAZ7 and SlJAZ11 (without a stop codon) were cloned into the Super1300-GFP vector via the *Hind* III and *Spe* I sites, which generated the SlJAZ7-GFP and SlJAZ11-GFP vectors, respectively. These constructs and the corresponding empty vectors were expressed in *N. benthamiana* leaves through *Agrobacterium* strain GV3101-mediated expression. GFP signals were captured after 50 h of infiltration with a confocal microscope (TCS-SP5, Leica, Wetzlar, Germany). The primers used in this experiment are shown in Table S2, see online supplementary material. These experiments were repeated three biological times.

### *Meloidogyne incognita* inoculation assays

T3 homozygous *SlWRKY45*-overexpressing plants, *Cas9*-free T2 homozygous *slwrky45* mutants, and their CM wild type plants were used for the experiments. Eggs of *M. incognita* were obtained from the infected tomato roots according to previously described methods [51], and incubated at 28°C to hatch J2s. When tomato seedlings grew to four true leaves, each plant was inoculated with approximately 400 J2s. At 7 d and 35 d after infection, the roots were soaked with 1.5% sodium hypochlorite for 5 min, washed with water twice, and stained by 3.5% acid fuchsin with 25% acetic acid. After staining, the roots were washed with water and decolored with the solution of glycerol, acetic acid, and H_2_O (1:1:1). Then, we weighed the root, and counted the number of galls on each. For counting the egg numbers, nematode eggs of each root were collected according to previously described methods [51], and the egg numbers were counted under a microscope (SMZ-140, Motic, Guangdong, China) in 20 aliquots of 10 μL. For Figs. 6C and S10 (see online supplementary material), the indicated plants were treated without or with 100 μM MeJA, and 24 h later, the plants were infected with *M. incognita*. These experiments were repeated three biological times.

### Quantification of JAs contents

H_2_JA (OlChemIm Ltd, Olomouc, Czech Republic) was used as the internal standard. Extraction and quantitative analysis of JA and JA-Ile contents was performed according to previously described methods [52]. Briefly, frozen roots were ground into powder. Then extraction buffer was added (2-propanol/H_2_O/concentrated HCl) and the internal standard, oscillated at 4°C for 30 min. After adding dichloromethane and centrifuging, the supernatants were evaporated to dryness under N_2_ gas, redissolved with methanol (0.1% formic acid), centrifuged, and filtrated using a 0.22 μM membrane. Finally, 2 μL of the solution was analysed using the ESI-HPLC-MS/MS system (HPLC, Agilent 1290, Agilent Technologies, CA, USA; MS, Applied Biosystems 6500 Quadrupole Trap, Applied Biosystem, CA, USA). These experiments were repeated three biological times.

### ChIP assays

ChIP assays were carried out in accordance with previously described protocols [53]. Briefly, 21-day-old CM and flag-*SlWRKY45-OE-5* plants were infected with the RKN *M. incognita* for 24 h. The harvested samples were soaked with 1% formaldehyde for cross-linking, neutralized with 0.125 M Glycine, and then ground into powder. The chromatin-protein was isolated, sonicated to cut the DNA to 300–500 bp, and then incubated with or without anti-flag-tag antibody (Agarose Conjugated) (Abmart, Shanghai, China) for 4 h at 4°C. The buffer with 0.1 M NaHCO_3_ and 0.5% SDS was used to elute the immunoprecipitated chromatin. Then the samples were added 5 M NaCl and incubated at 65°C overnight. Finally, DNA was extracted using a DNA extraction kit (GeneBette, Beijing, China). qPCR analysis was used to measure enrichment of promoter fragments by the % input method [54]. A fragment of the *SlACTIN2* promoter served as a negative control. The primers used for the experiment are listed in Table S6, see online supplementary material. These experiments were repeated three biological times.

### Dual-LUC assays

SlWRKY45, SlJAZ11, and GFP were ligated to the pGreenII 62-SK vector [55] via the *BamH* I and *EcoR* I sites. The ~1300 bp promoter of *SlAOC* was fused to the pGREENII 0800 LUC vector [55] via the *BamH* I and *Nco* I sites. *Agrobacterium* strains GV3101 (pSoup) carrying the corresponding constructs were cultured overnight, combined with the indicated recombinant pairs, incubated for 3–5 h, and co-infiltrated into *N. benthamiana* leaves. At 50 h after injection, LUC and REN activities were assessed with a dual-luciferase assay kit (E1910, Promega, WI, USA). The primers used for Dual-LUC assays are shown in Table S2, see online supplementary material. These experiments were repeated three biological times.

## Accession numbers

The accession numbers for genes are as follows: SlWRKY45 (Solyc08g067360), SlAOC (Solyc02g085730), SlLOXD (Solyc03g122340), SlJAZ1 (Solyc07g042170), SlJAZ2 (Solyc12g009220), SlJAZ3 (Solyc03g122190), SlJAZ4 (Solyc12g049400), SlJAZ5 (Solyc03g118540), SlJAZ6 (Solyc01g005440), SlJAZ7 (Solyc11g011030), SlJAZ8 (Solyc06g068930), SlJAZ9 (Solyc08g036640), SlJAZ10 (Soly08g036620), SlJAZ11 (Solyc08g036660), SlPDF (Solyc07g006380), SlPI-2 (Solyc03g020060), and SlACTIN2 (Solyc11g005330).

## Acknowledgements

This work was supported by the National Key R&D Program of China (2018YFD1000803), the National Natural Science Foundation of China (Grant No.31902026), Beijing Natural Science Foundation (Grant No.6194030), and Scientific Research Project of Beijing Municipal Commission of Education (Grant No.KM201910020013).

## Author contributions

S.W., S.S., and H.H. designed the research. H.H., W.Z., H.Q., C.L., L.S., R.Y., X.M., and J.M. performed the research. H.H., W.Z., and H.Q. analysed the data. S.W., S.S., and H.H. wrote the article.

## Data availability

All data generated in this study are available upon request.

## Conflict of interest

The authors declare no conflicts of interest.

## Supplementary data

Supplementary data is available at *Horticulture Research* online.

## Supplementary Material

Web_Material_uhac197
