# Supplementary material for: SlWRKY45 interacts with jasmonate-ZIM domain proteins to negatively regulate defense against the root-knot nematode Meloidogyne incognita in tomato
Source: Hortic Res. 2022 Sep 5;9:uhac197. doi: 10.1093/hr/uhac197 (PMC9630973; doi:10.1093/hr/uhac197)
Supplement: Web_Material_uhac197 [file web_material_uhac197.docx]

**
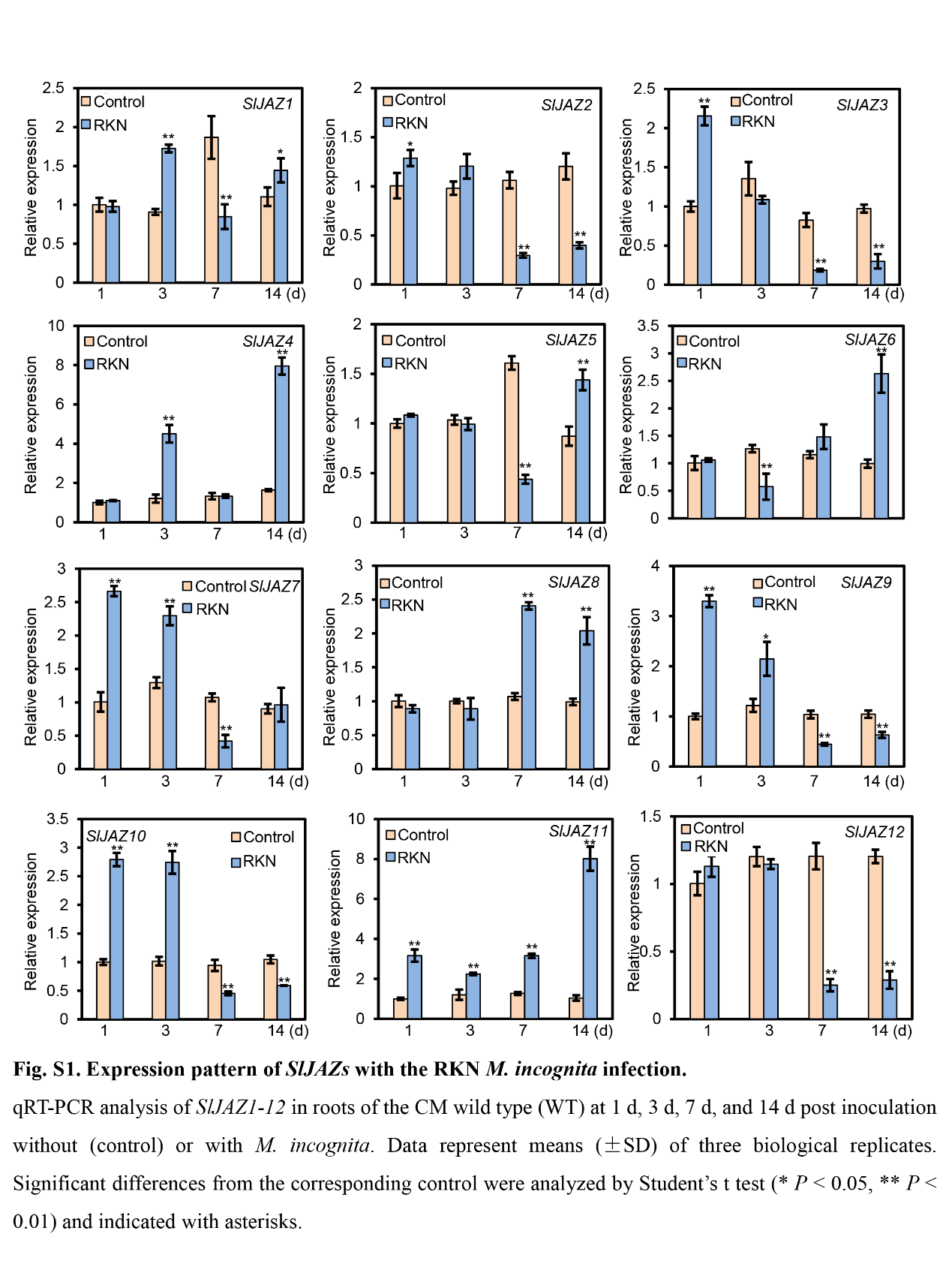
**

**Fig. S1. Expression pattern of *SlJAZs* with the RKN *M. incognita* infection.**

qRT-PCR analysis of *SlJAZ1-12* in roots of the CM wild type (WT) at 1 d, 3 d, 7 d, and 14 d post inoculation without (control) or with *M. incognita*. Data represent means (±SD) of three biological replicates. Significant differences from the corresponding control were analyzed by Student’s t test (* *P* < 0.05, ** *P* < 0.01) and indicated with asterisks.

**
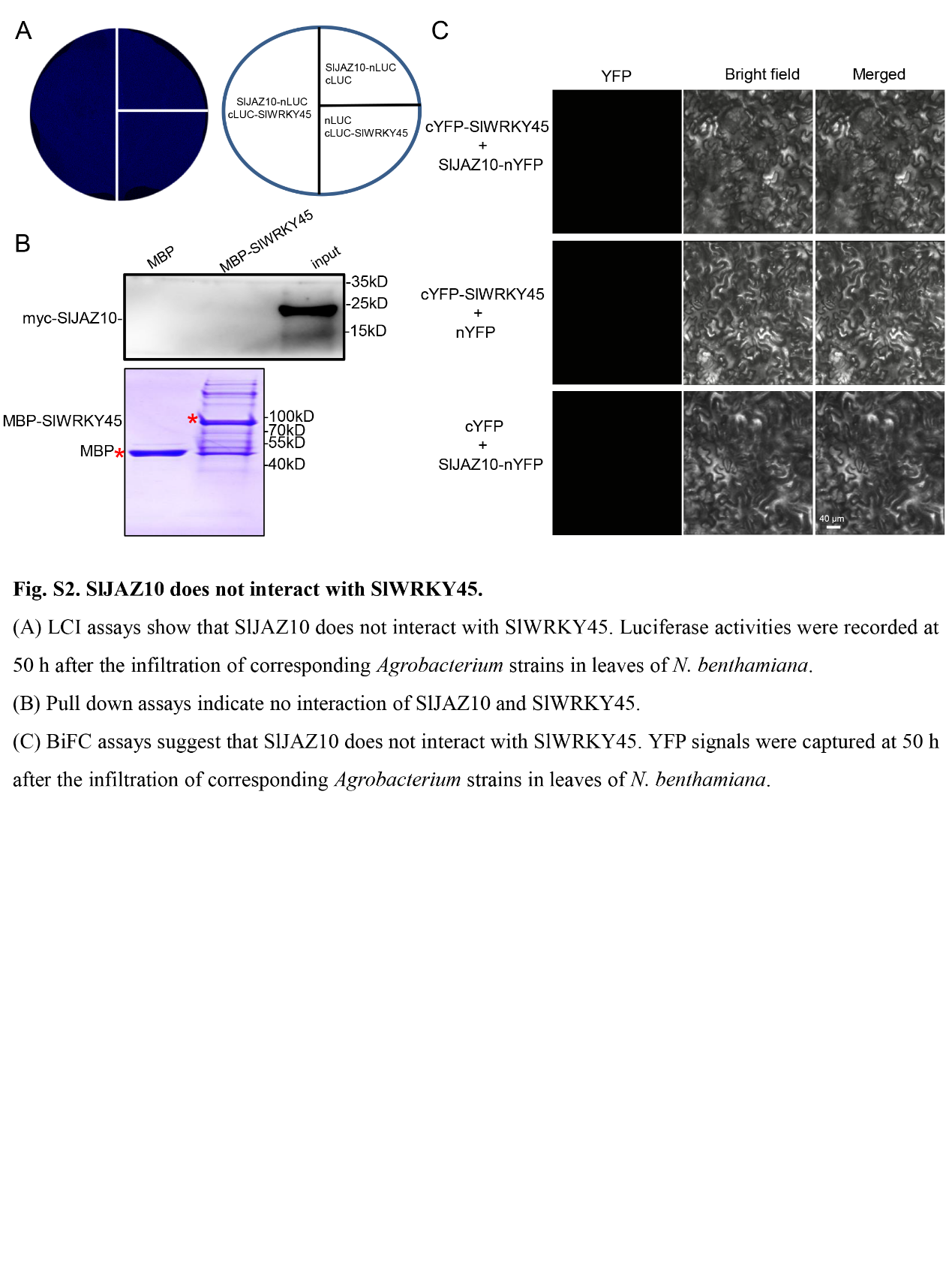
**

**Fig. S2. SlJAZ10 does not interact with SlWRKY45.**

(A) LCI assays show that SlJAZ10 does not interact with SlWRKY45. Luciferase activities were recorded at 50 h after the infiltration of corresponding *Agrobacterium* strains in leaves of *N. benthamiana*.

(B) Pull down assays indicate no interaction of SlJAZ10 and SlWRKY45.

(C) BiFC assays suggest that SlJAZ10 does not interact with SlWRKY45. YFP signals were captured at 50 h after the infiltration of corresponding *Agrobacterium* strains in leaves of *N. benthamiana*.

**
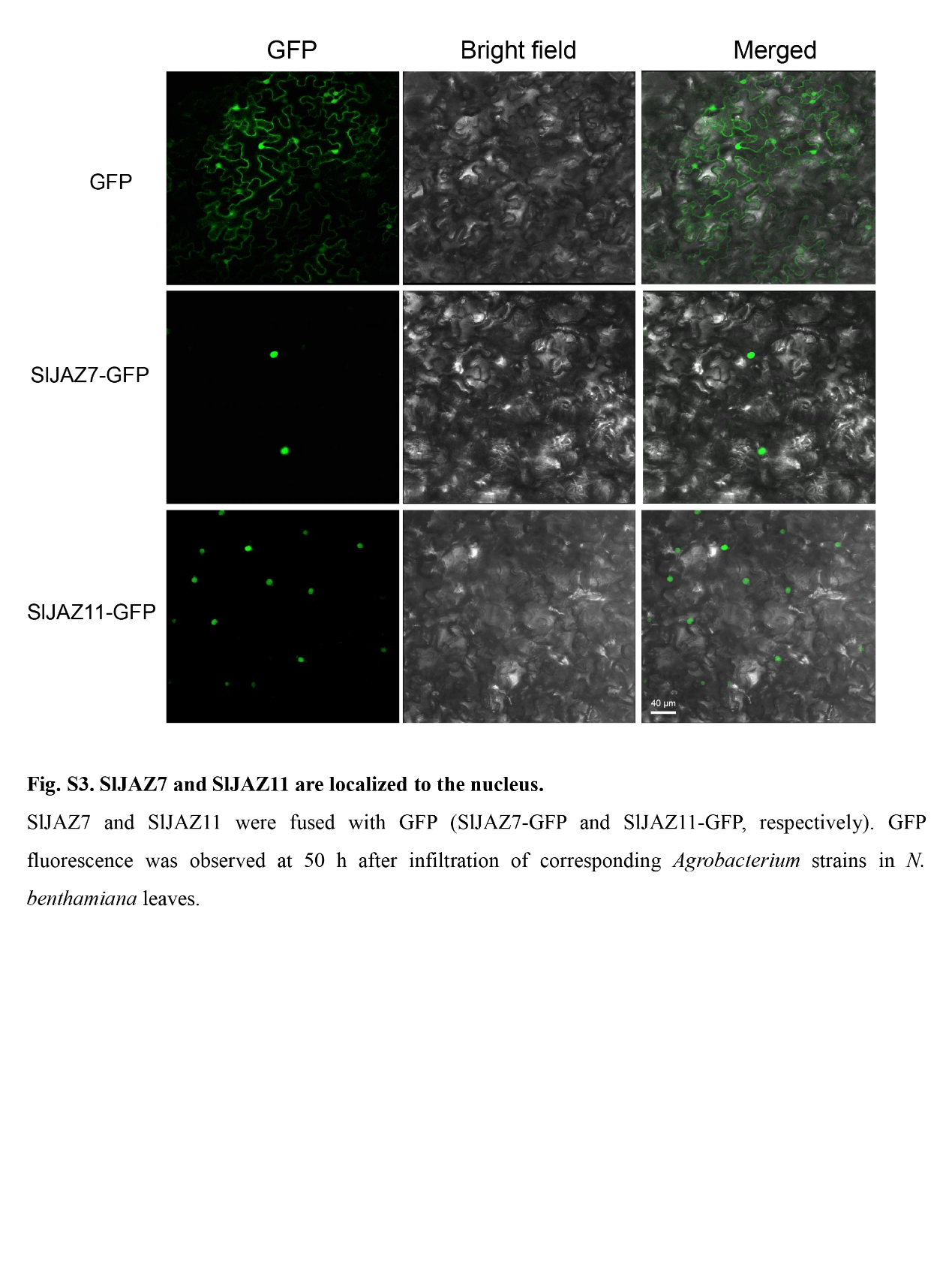
**

**Fig. S3. SlJAZ7 and SlJAZ11 are localized to the nucleus.**

SlJAZ7 and SlJAZ11 were fused with GFP (SlJAZ7-GFP and SlJAZ11-GFP, respectively). GFP fluorescence was observed at 50 h after infiltration of corresponding *Agrobacterium* strains in *N. benthamiana* leaves.

**
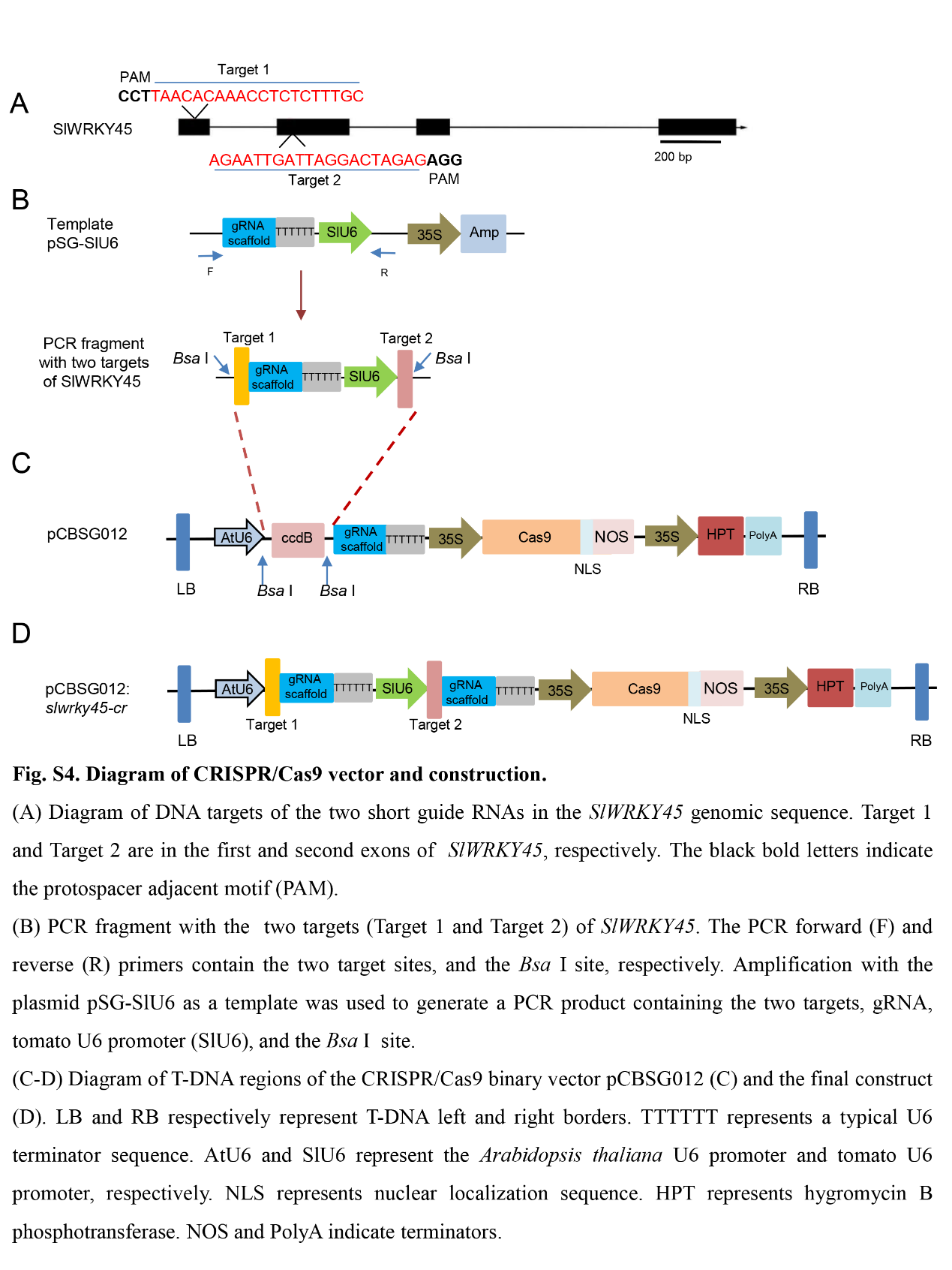
**

**Fig. S4. Diagram of CRISPR/Cas9 vector and construction.**

(A) Diagram of DNA targets of the two single guide RNAs in the *SlWRKY45* genomic sequence. Target 1 and Target 2 are in the first and second exons of *SlWRKY45*, respectively*.* The black bold letters indicate the protospacer adjacent motif (PAM).

(B) PCR fragment with the two targets (Target 1 and Target 2) of *SlWRKY45*. The PCR forward (F) and reverse (R) primers contain the two target sites, and the *Bsa* I site, respectively. Amplification with the plasmid pSG-SlU6 as a template was used to generate a PCR product containing the two targets, gRNA scaffold, tomato U6 promoter (SlU6), and the *Bsa* I site.

(C-D) Diagram of T-DNA regions of the CRISPR/Cas9 binary vector pCBSG012 (C) and the final construct (D). LB and RB respectively represent T-DNA left and right borders. TTTTTT represents a typical U6 terminator sequence. AtU6 and SlU6 represent the *Arabidopsis thaliana* U6 promoter and tomato U6 promoter, respectively. NLS represents nuclear localization sequence. HPT represents hygromycin B phosphotransferase. NOS and PolyA indicate terminators.

**
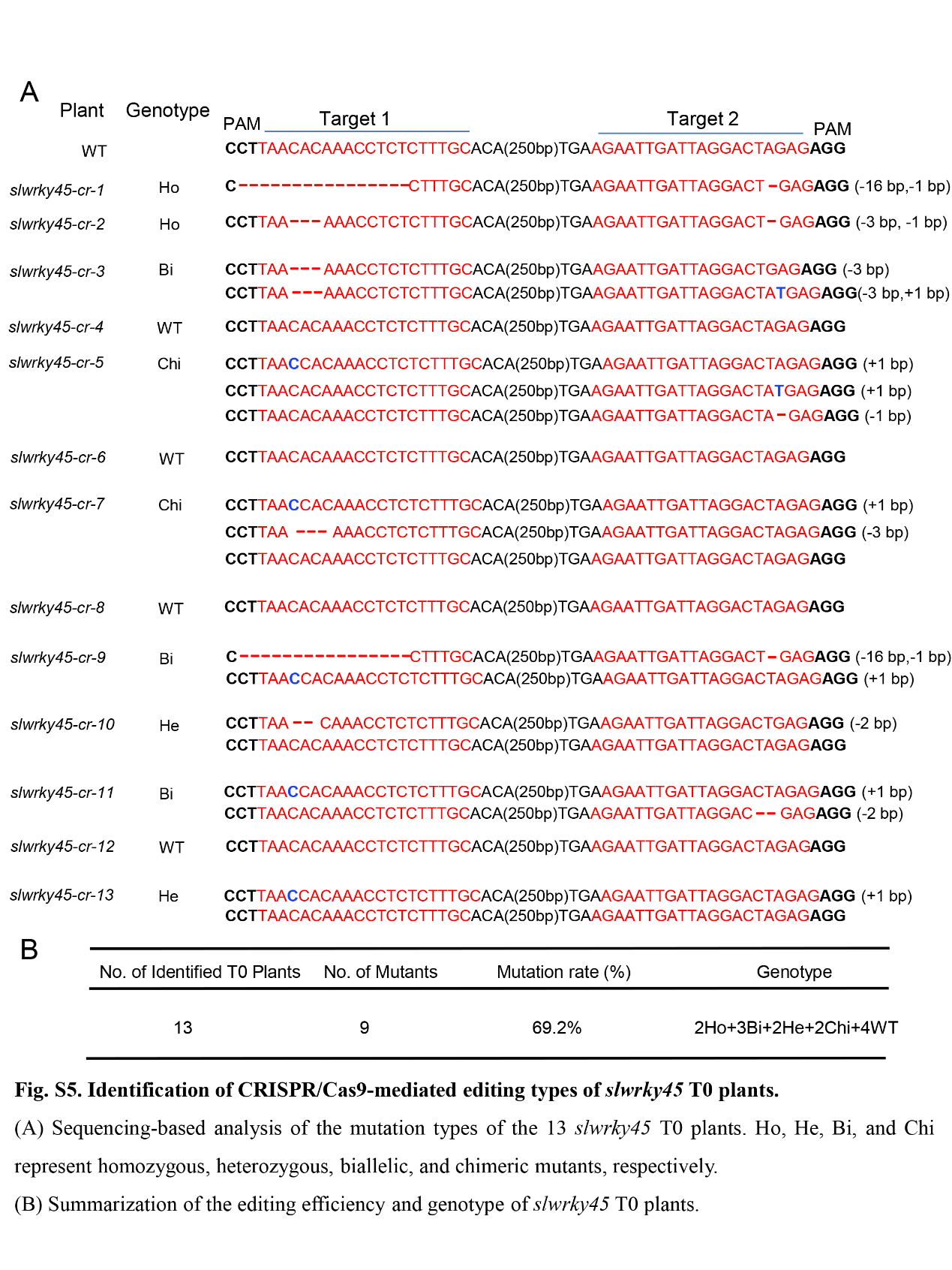
**

**Fig. S5. Identification of CRISPR/Cas9-mediated editing types of *slwrky45* T0 plants.**

(A) Sequencing-based analysis of the mutation types of the 13 *slwrky45* T0 plants. Ho, He, Bi, and Chi represent homozygous, heterozygous, biallelic, and chimeric mutants, respectively.

(B) Summarization of the editing efficiency and genotype of *slwrky45* T0 plants.

**
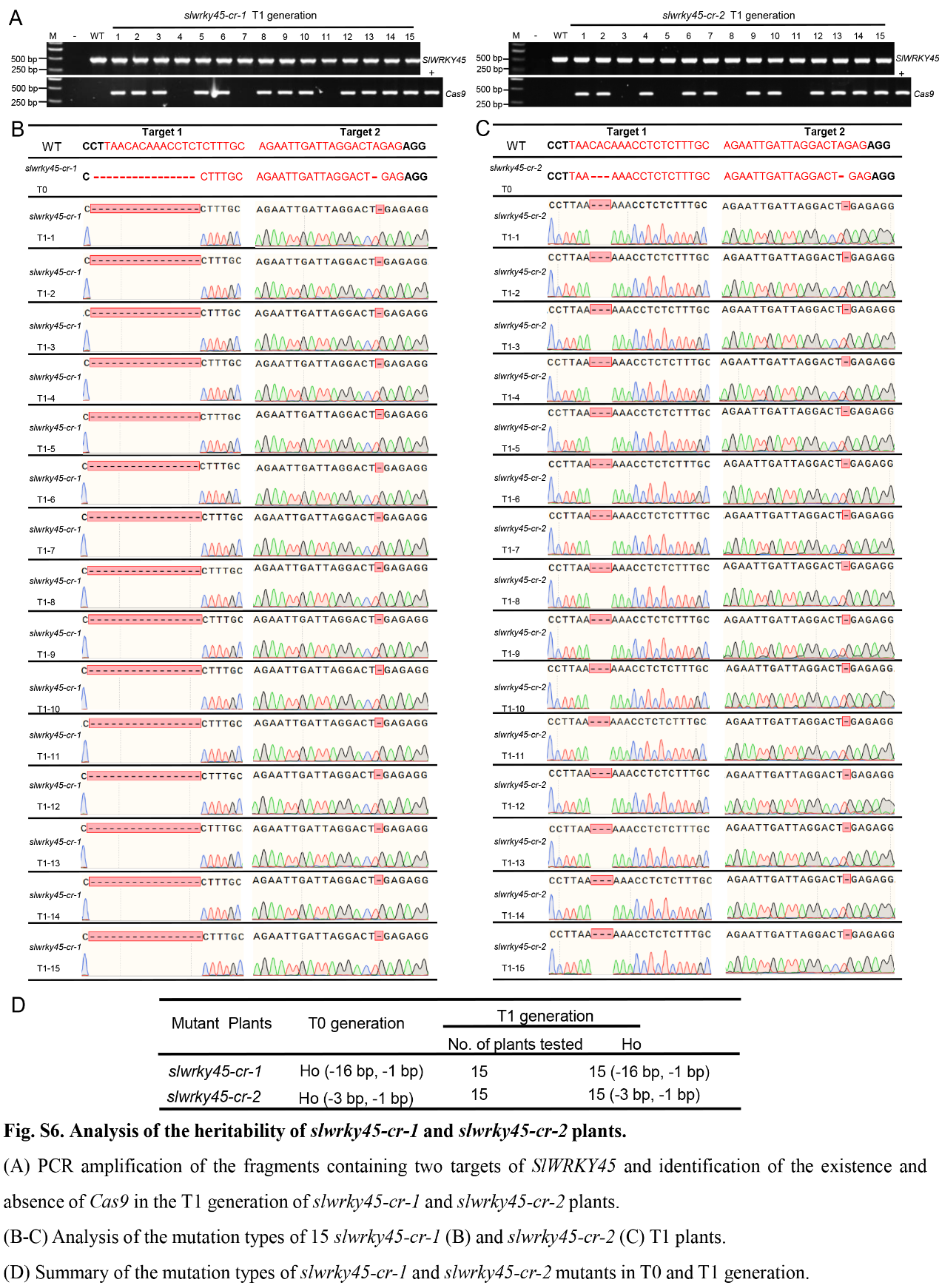
**

**Fig. S6. Analysis of the heritability of *slwrky45-cr-1* and *slwrky45-cr-2* plants.**

(A) PCR amplification of the fragments containing two targets of *SlWRKY45* and identification of the existence and absence of *Cas9* in the T1 generation of *slwrky45-cr-1* and *slwrky45-cr-2* plants.

(B-C) Analysis of the mutation types of 15 *slwrky45-cr-1* (B) and *slwrky45-cr-2* (C) T1 plants.

(D) Summary of the mutation types of *slwrky45-cr-1* and *slwrky45-cr-2* mutants in T0 and T1 generation.

**
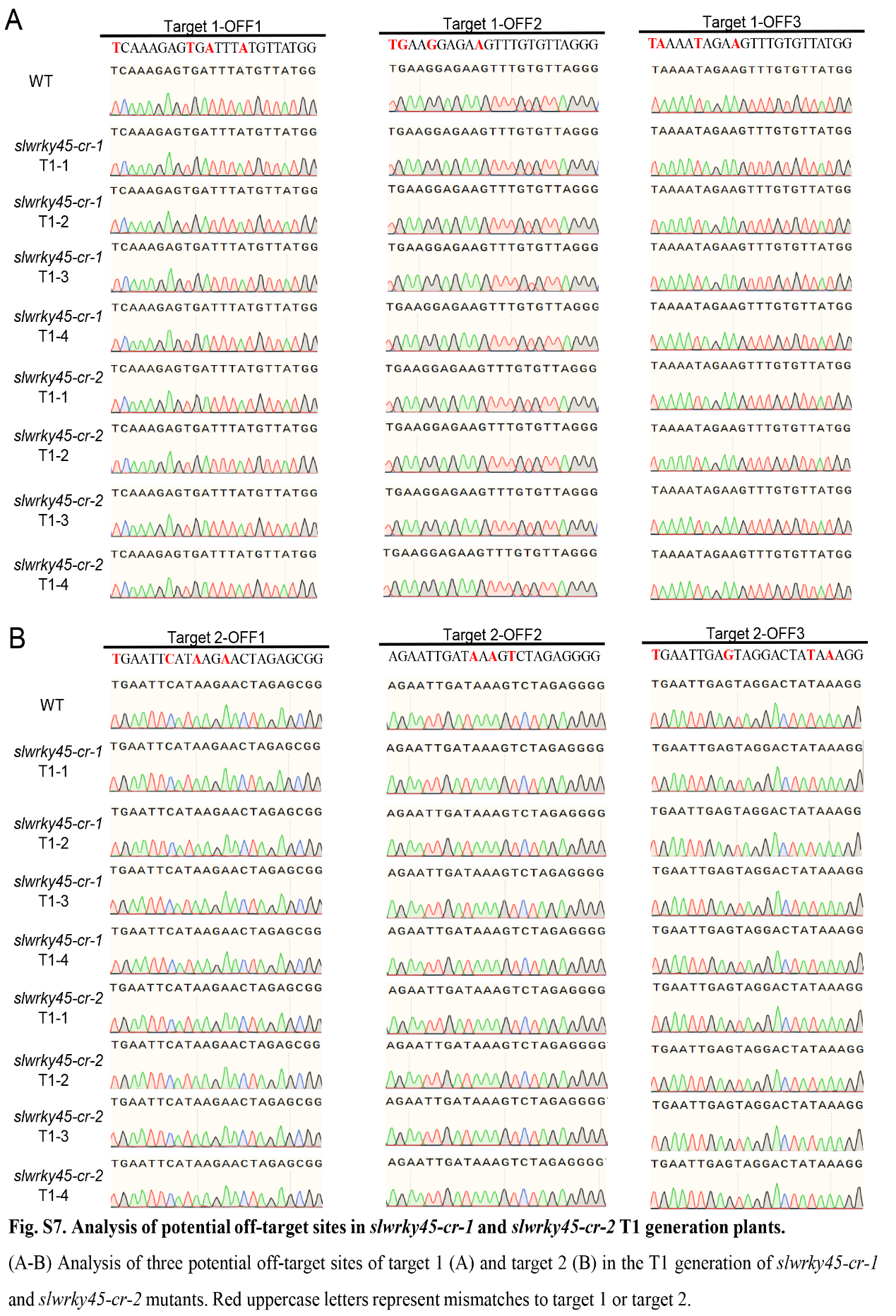
**

**Fig. S7. Analysis of potential off-target sites in *slwrky45-cr-1* and *slwrky45-cr-2* T1 generation plants.**

(A-B) Analysis of three potential off-target sites of target 1 (A) and target 2 (B) in the T1 generation of *slwrky45-cr-1* and *slwrky45-cr-2* mutants. Red uppercase letters represent mismatches to target 1 or target 2.

**
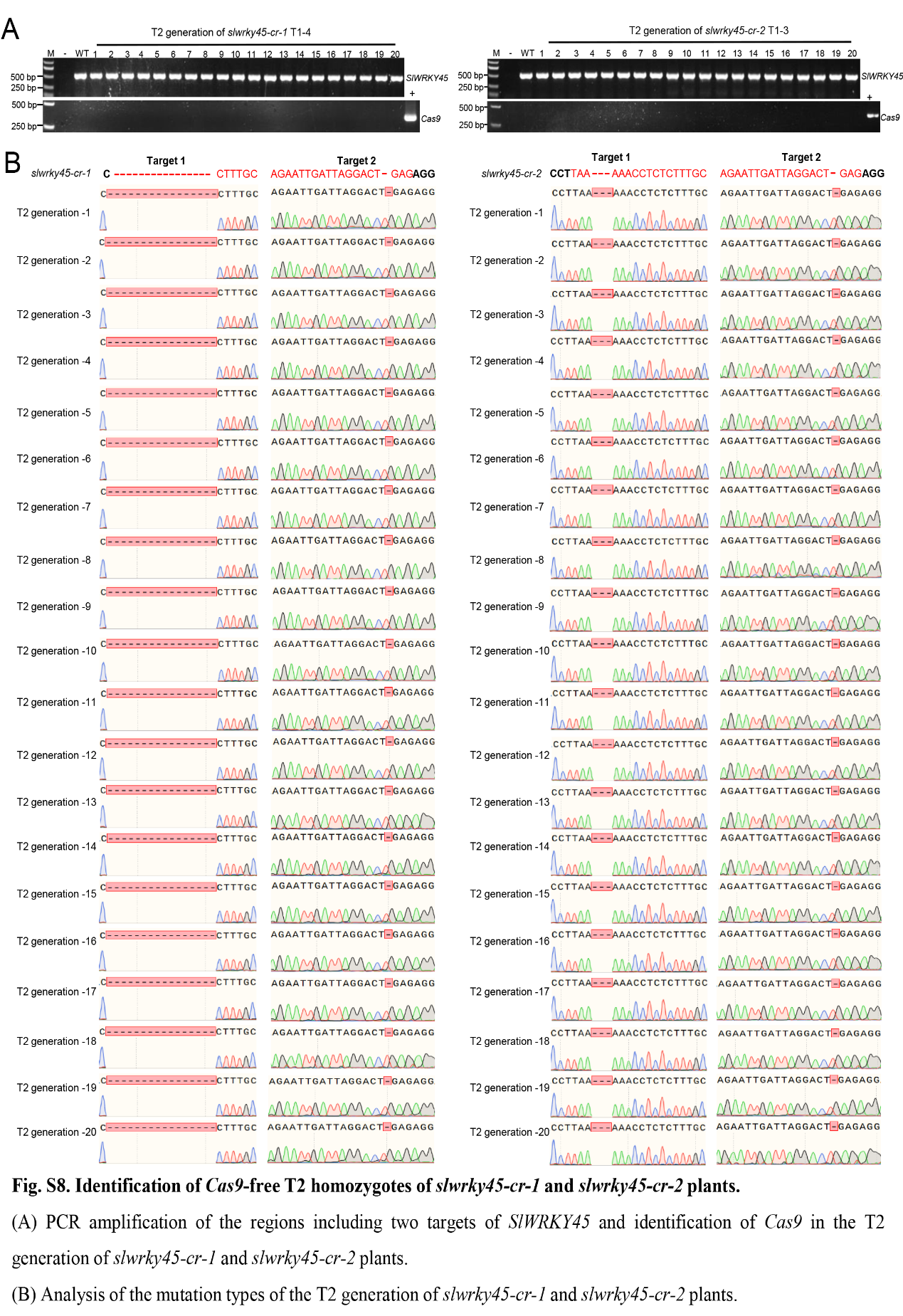
**

**Fig. S8. Identification of *Cas9*-free T2 homozygotes of *slwrky45-cr-1* and *slwrky45-cr-2* plants.**

(A) PCR amplification of the regions including two targets of *SlWRKY45* and identification of *Cas9* in the T2 generation of *slwrky45-cr-1* and *slwrky45-cr-2* plants.

(B) Analysis of the mutation types of the T2 generation of *slwrky45-cr-1* and *slwrky45-cr-2* plants.

**
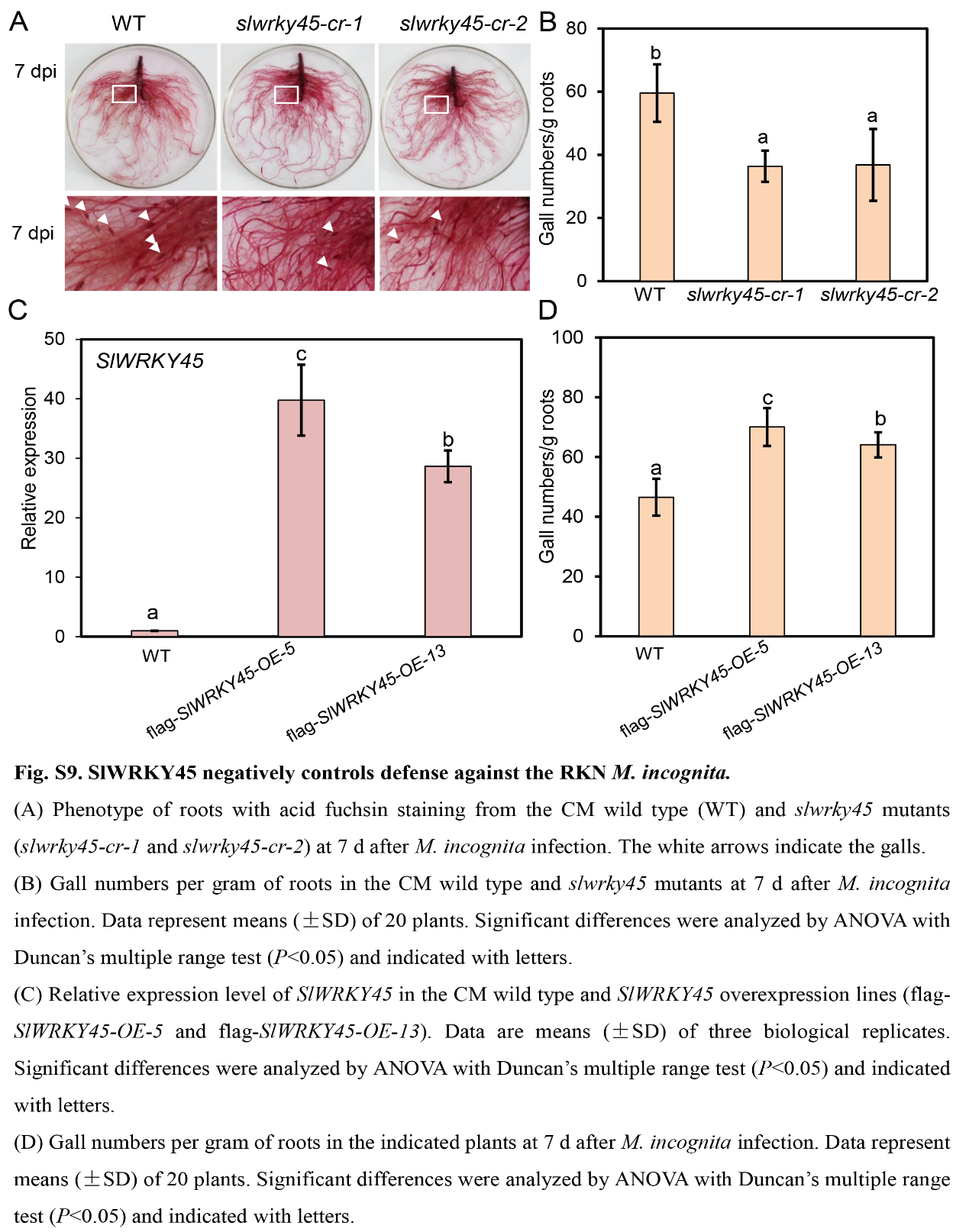
**

**Fig. S9. SlWRKY45 negatively controls defense against the RKN *M. incognita.***

(A) Phenotype of roots with acid fuchsin staining from the CM wild type (WT) and *slwrky45* mutants (*slwrky45-cr-1* and *slwrky45-cr-2*) at 7 d after *M. incognita* infection. The white arrows indicate the galls.

(B) Gall numbers per gram of roots in the CM wild type and *slwrky45* mutants at 7 d after *M. incognita* infection. Data represent means (±SD) of 20 plants. Significant differences were analyzed by ANOVA with Duncan’s multiple range test (*P*<0.05) and indicated with letters.

(C) Relative expression level of *SlWRKY45* in the CM wild type and *SlWRKY45* overexpression lines (flag-*SlWRKY45-OE-5* and flag-*SlWRKY45-OE-13*). Data are means (±SD) of three biological replicates. Significant differences were analyzed by ANOVA with Duncan’s multiple range test (*P*<0.05) and indicated with letters.

(D) Gall numbers per gram of roots in the indicated plants at 7 d after *M. incognita* infection. Data represent means (±SD) of 20 plants. Significant differences were analyzed by ANOVA with Duncan’s multiple range test (*P*<0.05) and indicated with letters.

**
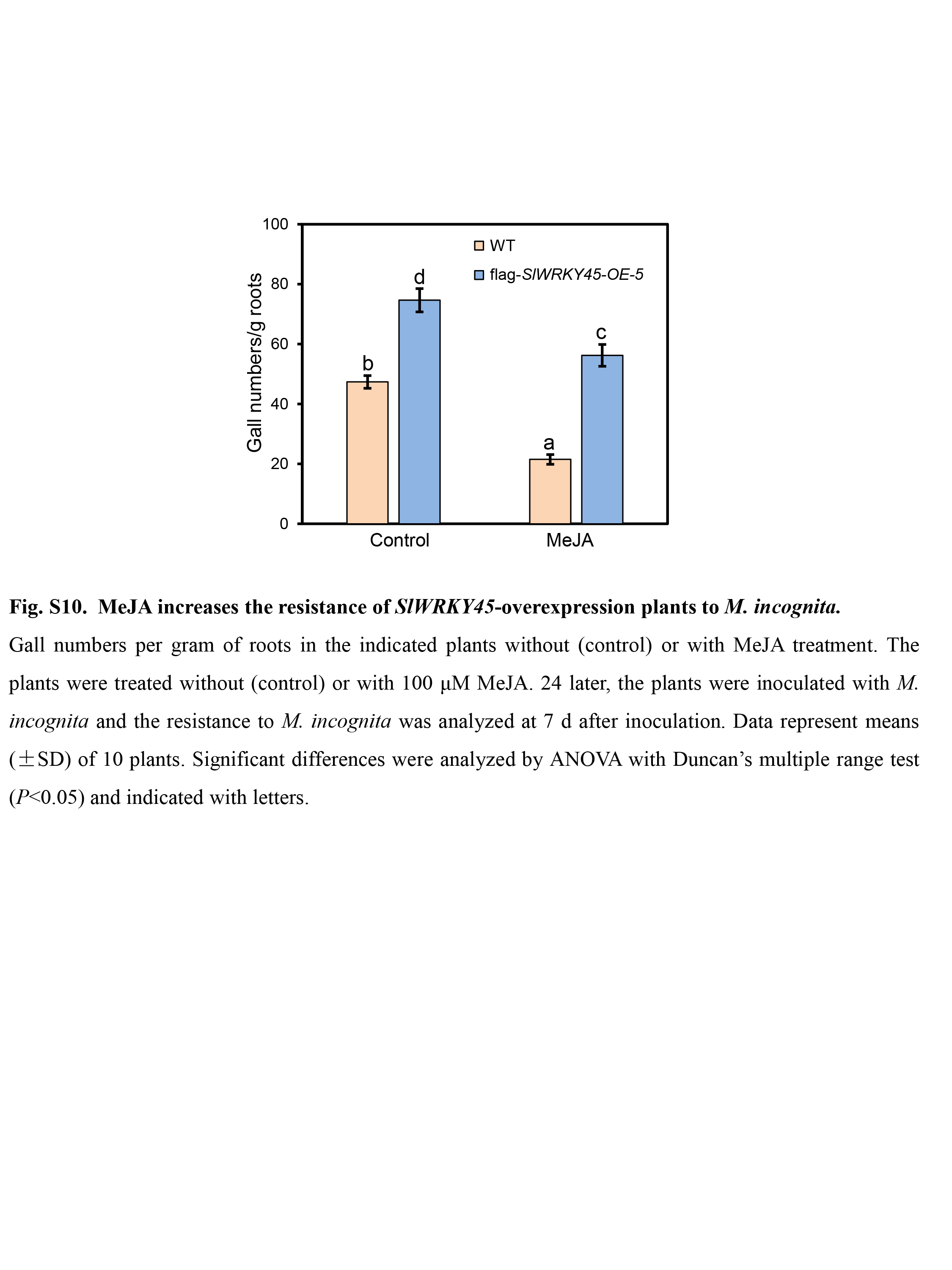
**

**Fig. S10. MeJA increases the resistance of *SlWRKY45*-overexpression plants to *M. incognita.***

Gall numbers per gram of roots in the indicated plants without (control) or with MeJA treatment. The plants were treated without (control) or with 100 μM MeJA. 24 later, the plants were inoculated with *M. incognita* and the resistance to *M. incognita* was analyzed at 7 d after inoculation. Data represent means (±SD) of 10 plants. Significant differences were analyzed by ANOVA with Duncan’s multiple range test (*P*<0.05) and indicated with letters.

**
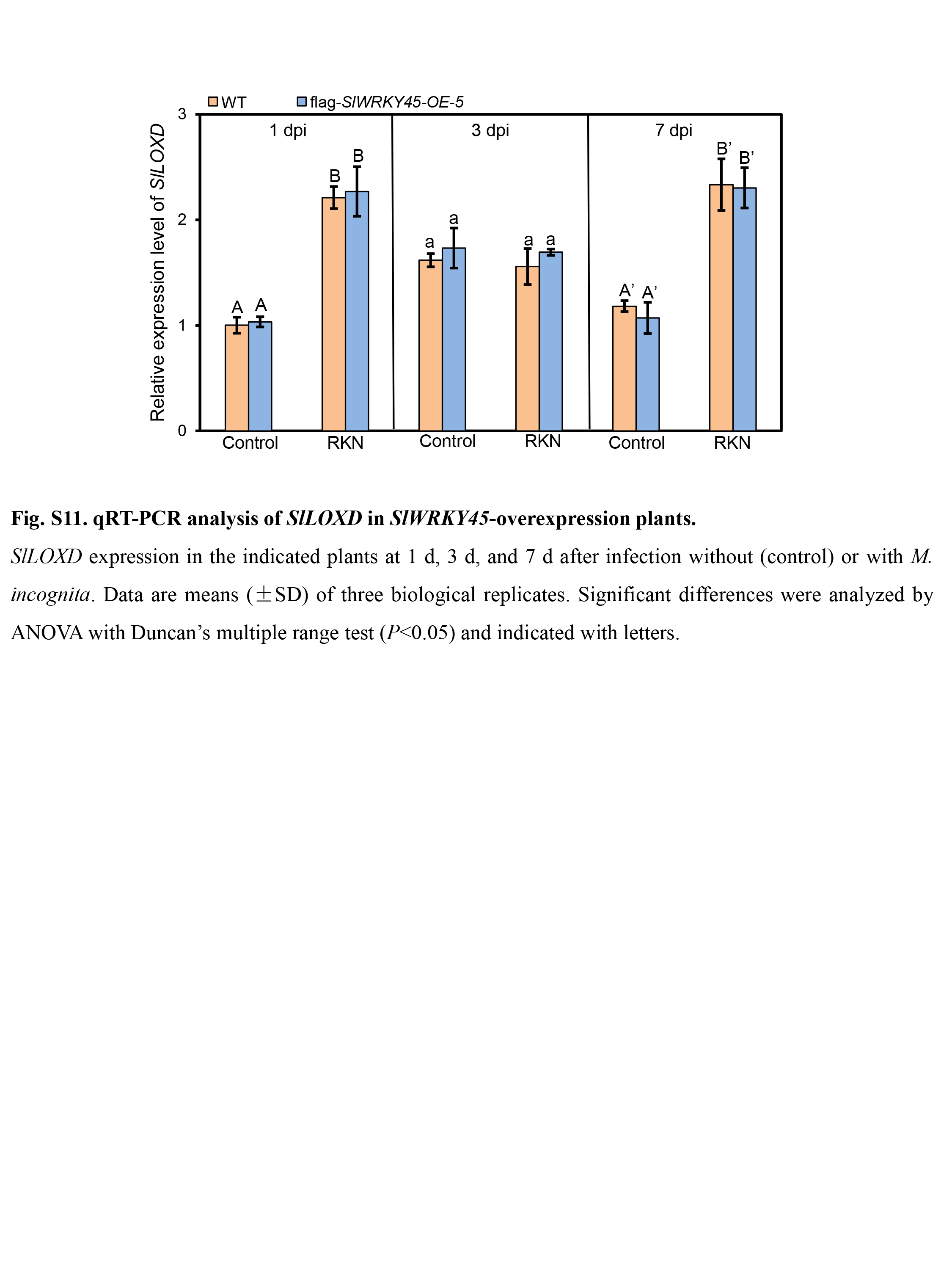
**

**Fig. S11. qRT-PCR analysis of *SlLOXD* in *SlWRKY45*-overexpression plants.**

*SlLOXD* expression in the indicated plants at 1 d, 3 d, and 7 d after infection without (control) or with *M. incognita*. Data are means (±SD) of three biological replicates. Significant differences were analyzed by ANOVA with Duncan’s multiple range test (*P*<0.05) and indicated with letters.

**Table S1. Analysis of the putative oﬀ-target sites in *slwrky45-cr-1* and *slwrky45-cr-2* plants.**

| Target | Name of putative off-target sites | Putative off-target locus | Sequence of the putative oﬀ-target site | No. of mismatching bases | No. of T1 transgenic lines sequenced | No.of plants with mutations |
| --- | --- | --- | --- | --- | --- | --- |
|  |  |  |  |  |  |  |
|  |  |  |  |  |  |  |
| Target 1 | OFF1 | SL2.50ch12:-5990796 | **T**CAAAGAG**T**G**A**TTT**A**TGTTATGG | 4 | 8 | 0 |
|  | OFF2 | SL2.50ch05:-37653553 | **TG**AA**G**GAGA**A**GTTTGTGTTAGGG | 4 | 8 | 0 |
|  | OFF3 | SL2.50ch02:+7025285 | **TA**AAA**T**AGA**A**GTTTGTGTTATGG | 4 | 8 | 0 |
| Target 2 | OFF1 | SL2.50ch09:-53722293 | **T**GAATT**C**AT**A**AG**A**ACTAGAGCGG | 4 | 8 | 0 |
|  | OFF2 | SL2.50ch01:+55181261 | AGAATTGAT**A**A**A**G**T**CTAGAGGGG | 3 | 8 | 0 |
|  | OFF3 | SL2.50ch12:+11202788 | **T**GAATTGA**G**TAGGACTA**T**A**A**AGG | 4 | 8 | 0 |

(Red uppercase letters represent the mismatching bases)

**Table S2. Primers used for vector construction.**

| **Assay** | **Primer** | **Sequence (5’-3’)** |
| --- | --- | --- |
| **Y2H** | SlWRKY45-BD/AD-F | GGGGAATTCATGGATACAAACTTGGGAGAC |
|  | SlWRKY45-BD/AD-R | CCGCTCGAGTTAATTGAAAGGCAAATCATATTC |
|  | SlJAZ1-AD/BD-F | GGGGAATTCATGGCTTCATCGGAGATTGTGG |
|  | SlJAZ1-AD/BD-R | CCGCTCGAGCTAGTATTGCTCAGTTTTCACT |
|  | SlJAZ2-AD/BD-F | CCCCCCATGGATGGGGTCATCGGAAAATATGG |
|  | SlJAZ2-AD/BD-R | CCGCTCGAGCTAGAAATATTGCTCAGTTTTAAC |
|  | SlJAZ3-AD/BD-F | GGGGAATTCATGTCGAATTTATGTGACGCTC |
|  | SlJAZ3-AD/BD-R | ACGCGTCGACCTATAACTTGAAATTGAGATC |
|  | SlJAZ4-AD/BD-F | GGGGAATTCATGTCAAATAGGCAACTTTGTTC |
|  | SlJAZ4-AD/BD-R | CCGCTCGAGCTAGAAATTGAGATCAAAATG |
|  | SlJAZ5-AD/BD-F | AGCCAATTGATGGAGAGAGATTTCATGGGGTTG |
|  | SlJAZ5-AD/BD-R | ACGCGTCGACCTACTTGACCAAACTGATTATG |
|  | SlJAZ6-AD-F | AGCCAATTGATGGAGAGGGACTTTATGGGATTG |
|  | SlJAZ6-AD-R | CCGCTCGAGCTAGGTCTCCTTACCGGCTAAC |
|  | SlJAZ7-AD/BD-F | GGGGAATTCATGGATTCAAGAATGGAGATAG |
|  | SlJAZ7-AD/BD-R | CCGCTCGAGTTAGTTTTCCCAATGAACGCTTG |
|  | SlJAZ8-AD-F | GGGGAATTCATGCATTGGTCATATTCTAAC |
|  | SlJAZ8-AD-R | CCGCTCGAGCTAATTTGCAGCAGGAAGTGAAG |
|  | SlJAZ9-AD-F | GGGGAATTCATGAGAAGAAATTGTAATTTGG |
|  | SlJAZ9-AD-R | CCGCTCGAGCTATTTGTGATATGGCGAAGTTG |
|  | SlJAZ10-AD-F | CCCCCCATGGATGAGAAGAAAGTGTAATTTGG |
|  | SlJAZ10-AD-R | CCGCTCGAGCTAGTGATGATATGGAGAAG |
|  | SlJAZ11-AD/BD -F | GGGGAATTCATGAGAAGAAATTGTAATTTG |
|  | SlJAZ11-AD/BD -R | CCGCTCGAGCTAGTGATGATATGGCGAAG |
|  | SlJAZ7NT-AD-F | GGGGAATTCATGGATTCAAGAATGGAGATAG |
|  | SlJAZ7NT-AD-R | ACGCGTCGACAGCATAATTTGTGGATGCATTTG |
|  | SlJAZ7CT-AD-F | CGGGGTACCATGAATAAAGAAAAATATGAAG |
|  | SlJAZ7CT-AD-R | CCGCTCGAGTTAGTTTTCCCAATGAACGCTTG |
| **LCI** | SlJAZ11NT-AD-F | GGGGAATTCATGAGAAGAAATTGTAATTTG |
|  | SlJAZ11NT-AD-R | CCGCTCGAGCTATTGTAATAATGGTGATGATGG |
|  | SlJAZ11CT-AD-F | GGGGAATTCATGGAAACAGAGGAGAAAACAAAC |
|  | SlJAZ11CT-AD-R | CCGCTCGAGCTAGTGATGATATGGCGAAG |
|  | SlJAZ1-nLUC-F | CGGGGTACCATGGCTTCATCGGAGATTGTGG |
|  | SlJAZ1-nLUC-R | ACGCGTCGACGTATTGCTCAGTTTTCACT |
|  | SlJAZ2-nLUC-F | CGGGGTACCATGGGGTCATCGGAAAATATGG |
|  | SlJAZ2-nLUC-R | ACGCGTCGACGAAATATTGCTCAGTTTTAACAAATTG |
|  | SlJAZ3-nLUC-F | CGGGGTACCATGTCGAATTTATGTGACGCTC |
|  | SlJAZ3-nLUC-R | ACGCGTCGACTAACTTGAAATTGAGATC |
|  | SlJAZ4-nLUC-F | CGGGGTACCATGTCAAATAGGCAACTTTGTTC |
|  | SlJAZ4-nLUC-R | ACGCGTCGACGAAATTGAGATCAAAATG |
|  | SlJAZ7-nLUC-F | CGGGGTACCATGGATTCAAGAATGGAGATAG |
|  | SlJAZ7-nLUC-R | ACGCGTCGACGTTTTCCCAATGAACGCTTG |
|  | SlJAZ10-nLUC-F | CGGGGTACCATGAGAAGAAAGTGTAATTTGG |
|  | SlJAZ10-nLUC-R | ACGCGTCGACGTGATGATATGGAGAAG |
|  | SlJAZ11-nLUC-F | CGGGGTACCATGAGAAGAAATTGTAATTTG |
|  | SlJAZ11-nLUC-R | ACGCGTCGACGTGATGATATGGCGAAGTTG |
|  | SlJAZ7NT-nLUC-F | CGGGGTACCATGGATTCAAGAATGGAGATAG |
|  | SlJAZ7NT-nLUC-R | ACGCGTCGACAGCATAATTTGTGGATGCATTTG |
|  | SlJAZ11NT-nLUC-F | CGGGGTACCATGAGAAGAAATTGTAATTTG |
|  | SlJAZ11NT-nLUC-R | ACGCGTCGACTTGTAATAATGGTGATGATGG |
|  | cLUC-SlWRKY45-F | CGGGGTACCATGGATACAAACTTGGGAGAC |
|  | cLUC-SlWRKY45-R | ACGCGTCGACTTAATTGAAAGGCAAATCATATTC |
| **Pull down** | myc-SlJAZ7-F | AGACCCGGGATGGATTCAAGAATGGAGATAG |
|  | myc-SlJAZ7-R | ATCGAGCTCTTAGTTTTCCCAATGAACGCTTG |
|  | myc-SlJAZ10-F | AGACCCGGGATGAGAAGAAAGTGTAATTTGG |
|  | myc-SlJAZ10-R | ATCGAGCTCCTAGTGATGATATGGAGAAG |
|  | myc-SlJAZ11-F | AGACCCGGGATGAGAAGAAATTGTAATTTG |
|  | myc-SlJAZ11-R | ATCGAGCTCCTAGTGATGATATGGCGAAG |
|  | MBP-SlWRKY45-F | ACGCGTCGAC ATGGATACAAACTTGGGAGAC |
|  | MBP-SlWRKY45-R | CCGGAATTCTTAGTGGTGGTGGTGGTGGTGATTG  AAAGGCAAATCATATTCG |
| **BiFC** | SlJAZ7-nYFP-F | CGGGGTACCATGGATTCAAGAATGGAGATAG |
|  | SlJAZ7-nYFP-R | AAAGGGCCCCGTTTTCCCAATGAACGCTTG |
|  | SlJAZ10-nYFP-F | CGGGGTACCATGAGAAGAAAGTGTAATTTGG |
|  | SlJAZ10-nYFP-R | AAAGGGCCCCGTGATGATATGGAGAAG |
|  | SlJAZ11-nYFP-F | CGGGGTACCATGAGAAGAAATTGTAATTTG |
|  | SlJAZ11-nYFP-R | AAAGGGCCCCGTGATGATATGGCGAAGTTG |
|  | cYFP-SlWRKY45-F | CGGGGTACCATGGATACAAACTTGGGAGAC |
|  | cYFP-SlWRKY45-R | AAAGGGCCCCATTGAAAGGCAAATCATATTC |
| **Subcellular**  **localization** | GFP-SlWRKY45 -F | GGGGAATTCATGGATACAAACTTGGGAGAC |
|  | GFP-SlWRKY45- R | CGCGGATCCTTAATTGAAAGGCAAATCATATTC |
|  | SlJAZ7-GFP-F | CGGAAGCTTATGGATTCAAGAATGGAGATAG |
|  | SlJAZ7-GFP-R | CGGACTAGTGTTTTCCCAATGAACGCTTG |
|  | SlJAZ11-GFP-F | CGGAAGCTTATGAGAAGAAATTGTAATTTG |
|  | SlJAZ11-GFP-R | CGGACTAGTGTGATGATATGGCGAAGTTGTTTG |
| **Dual-LUC** | SlWRKY45-pGreenII 62-SK-F | CGCGGATCCATGGATACAAACTTGGGAGAC |
|  | SlWRKY45-pGreenII 62-SK-R | GGGGAATTCTTAATTGAAAGGCAAATCATATTC |
|  | SlJAZ11-pGreenII 62-SK-F | CGCGGATCCATGAGAAGAAATTGTAATTTG |
|  | SlJAZ11-pGreenII 62-SK-R | GGGGAATTCCTAGTGATGATATGGCGAAG |
|  | GFP-pGreenII 62-SK-F | CGCGGATCCATGGTGAGCAAGGGCGAGGAGC |
|  | GFP-pGreenII 62-SK-R | GGGGAATTCTTACTTGTACAGCTCGTCCATGC |
|  | SlAOCpro-pGREEN0800-F | CGCGGATCCTTCTTTTTTAAGTTAGTGTCAAAGTTAGTGTCACGTAGATCG |
|  | SlAOCpro-pGREEN0800-R | CCCCCCATGGTGTATAAGTATTAAAGAAGTGACATC |
| **Construction of CRISPR/Cas9 vector** | SlWRKY45-T1-F | CAATGGTCTCATGATTGCAAAGAGAGGTTTGTGTTAGTTTTAGAGCTAGAAATA |
|  | SlWRKY45-T2-R | TTGGGGTCTCTAAACCTCTAGTCCTAATCAATTCC AAACTACACTGTTAGATTT |
| **Construction of SlWRKY45-overexpression vector** | flag-SlWRKY45-OE-F | ACGCGTCGACATGGATACAAACTTGGGAGAC |
|  | flag-SlWRKY45-OE-R | CGGACTAGTTTAATTGAAAGGCAAATCATATTC |

**Table S3. Primers used for analysis of the target site mutation.**

| **Target site** | **Primer** | **Sequence (5’-3’)** |
| --- | --- | --- |
| SlWRKY45-T1, T2 | For | CCATTTCTTCCCAATAACAACAAC |
|  | Rev | TTTGCAGCAATTCAATTAGGTGAG |
|  | Seq | CCATTTCTTCCCAATAACAACAAC |

**Table S4. Primers used for analysis of the existence of *Cas9*.**

| **Primer** | **Sequence (5’-3’)** |
| --- | --- |
| Cas9-F | GACAAGAAGTACAGCATCGG |
| Cas9-R | CTCGTGGTAGGCCACCTCG |

**Table S5. Primers used for analysis of off-target site mutation.**

| **Off-target site** | **Primer** | **Sequence (5’-3’)** |
| --- | --- | --- |
| SlWRKY45-target 1  off-target site 1 | For | TGAGTCTAAGAATGGCATAATT |
|  | Rev | GGTAAGGGTTTCATCAGGCCAAG |
|  | Seq | GGTAAGGGTTTCATCAGGCCAAG |
| SlWRKY45-target 1  off-target site 2 | For | GCTCTTGTTCAGTCACAGTGTAGTTC |
|  | Rev | GCCAAAGTTGAGGTGATAGAGAG |
|  | Seq | GCCAAAGTTGAGGTGATAGAGAG |
| SlWRKY45-target 1  off-target site 3 | For | CGATTAATTGTCCATTCCCTCT |
|  | Rev | TTGGGCGGGGAACCCGAGGAA |
|  | Seq | CGATTAATTGTCCATTCCCTCT |
| SlWRKY45-target 2  off-target site 1 | For | ACGGTGTTGCTATGGTTGGA |
|  | Rev | GTAACAGTGAACCTGTTAAG |
|  | Seq | GTAACAGTGAACCTGTTAAG |
| SlWRKY45-target 2  off-target site 2 | For | AAGTGCCATTCACGGACGAAT |
|  | Rev | TCATTATTAAAGTCTGCATCGCG |
|  | Seq | AAGTGCCATTCACGGACGAAT |
| SlWRKY45-target 2  off-target site 3 | For | ACCTTCCCAAATAATCATATC |
|  | Rev | TAACTCTTTCACTGGTTTGAGA |
|  | Seq | ACCTTCCCAAATAATCATATC |

**Table S6. Primers used for qRT-PCR analysis.**

| SlJAZ1-qPCR-F | TTCCCTCAAGGTGGAATGAAGGCT |
| --- | --- |
| SlJAZ1-qPCR-R | TCCGAAACTCGGAACCACCAAATC |
| SlJAZ2-qPCR-F | ACCTGATCAACCAGAGAAGGCA |
| SlJAZ2-qPCR-R | AAACTCACACCAGATTGATCAGCTGT |
| SlJAZ3-qPCR-F | TTCCCTGCTGACAAAGCTAGAGCA |
| SlJAZ3-qPCR-R | AGGGTGCAGATGAAACTGATCCGA |
| SlJAZ4-qPCR-F | GCCAAAGCCTCAGCAACAAAGGAT |
| SlJAZ4-qPCR-R | ATCACTGCTCTGGCTTTCTCTGCT |
| SlJAZ5-qPCR-F | TCAGCTGTTCCGTCTAGCAGCATT |
| SlJAZ5-qPCR-R | TGCATTTGGTGTAACAGGTGGTGC |
| SlJAZ6-qPCR-F | AGTCGATGCTGGTCTCAAACGTCA |
| SlJAZ6-qPCR-R | TCGAAGACATTGACCATCCCACCA |
| SlJAZ7-qPCR-F | TTGCTATGGCTCGTAGAGCAACTC |
| SlJAZ7-qPCR-R | TTTCCCAATGAACGCTTGACGACG |
| SlJAZ8-qPCR-F | TCGTCAACCTCCCAATCATAAC |
| SlJAZ8-qPCR-R | GGAAAGGGTAGTGAGTGCATC |
| SlJAZ9-qPCR-F | TTTGGAGCTCACTCTTATGCCTCC |
| SlJAZ9-qPCR-R | AGCTCAGTAGCATCGGAAACCACA |
| SlJAZ10-qPCR-F | GGAACTCACTCTTTCTCCTAGCAAC |
| SlJAZ10-qPCR-R | TGGTGATGAAGGCTCAGACAGCTT |
| SlJAZ11-qPCR-F | GGAGTTTAGGCTTATGCCACCTTC |
| SlJAZ11-qPCR-R | GGCTCAGATATTGGTGACAGACTC |
| SlJAZ12-qPCR-F | TGCGCATTCCGAGGCATGATGATA |
| SlJAZ12-qPCR-R | CCTTCTTGCAATTGGCAACTCTGCT |
| SlWRKY45-qPCR-F | CTATTGACCTTAACACAAACC |
| SlWRKY45-qPCR-R | GTTACTAACTTCTTGTTCTCC |
| SlPDF-qPCR-F | GCAAAGCACCAAGCCAAAC |
| SlPDF-qPCR-R | GCATAGACACTTCCTTTGG |
| SlPI-2-qPCR-F | CCTATTCAAGATGTCCCCGTTC |
| SlPI-2-qPCR-R | GGGCAATCCAGAAGATGG |
| SlAOC-qPCR-F | ACTTACTACCACCTCTAC |
| SlAOC-qPCR-R | AGTGTTAGTTGAATCTGTT |
| SlLOXD-qPCR-F | CCGTGGTTGACACATTATCG |
| SlLOXD-qPCR-R | ACAGCAGTCCGCCCTATTTA |
| pSlAOC-1-qPCR-F | GCTTCACAAGCTTATATGT |
| pSlAOC-1-qPCR-R | GTCTTATTATTAATTGTTACA |
| pSlAOC-2-qPCR-F | GGCCTGCGCAGGAGAAACACG |
| pSlAOC-2-qPCR-R | GGTGTTAGATGTGATGATGGTAG |
| pSlAOC-3-qPCR-F | CCTAGAGCGATAGAAAATTTT |
| pSlAOC-3-qPCR-R | GAAAATATGTGTGTCTAACTCC |
| pSlActin2-qPCR-F | CGAGAAGTACGTGATGCAGTGT |
| pSlActin2-qPCR-R | CCAGTCCAGATACCTAGTCAGC |
| SlActin2-qPCR-F | TTGCTGACCGTATGAGCAAG |
| SlActin2-qPCR-R | GGACAATGGATGGACCAGAC |
